# Initiating Injectable Buprenorphine in People Hospitalized With Infections: A Randomized Clinical Trial

**DOI:** 10.1001/jamanetworkopen.2025.13000

**Published:** 2025-05-30

**Authors:** Nikhil Seval, Prerana Roth, Cynthia A. Frank, Angela Di Paola, Alain H. Litwin, Brent Vander Wyk, Victor Neirinckx, Esther Schlossberg, Patrick Lawson, Michelle Strong, Meredith A. Schade, Jonathan Nunez, Frances R. Levin, Kathleen T. Brady, Edward V. Nunes, Sandra A. Springer

**Affiliations:** 1 Yale AIDS Program, Section of Infectious Disease, Department of Internal Medicine, Yale School of Medicine, New Haven, Connecticut; 2 College of Physicians and Surgeons of Columbia University, New York, New York; 3 Addiction Medicine Center, Prisma Health, Greenville, South Carolina; 4 University of South Carolina School of Medicine Greenville; 5 Department of Psychiatry and Behavioral Sciences, Medical University of South Carolina, Charleston; 6 Division of Infectious Diseases, Department of Medicine, Penn State Milton S. Hershey Medical Center, Hershey, Pennsylvania; 7 Division of Infectious Diseases and HIV Medicine, Drexel University College of Medicine, Philadelphia, Pennsylvania; 8 The New York State Psychiatric Institute, New York, New York

## Abstract

**Importance:**

Hospitalizations are increasing in the US due to infections related to opioid use disorder (OUD); however, few patients have treatment with medications for OUD (MOUD) initiated. Injectable long-acting buprenorphine (LAB) could help improve MOUD receipt and infection treatment completion.

**Objective:**

To compare initiation of LAB combined with infectious disease (ID) management (ID-LAB) with treatment as usual (TAU) during inpatient medical hospitalization periods for improving receipt of MOUD at 12 weeks.

**Design, Setting, and Participants:**

The Coordinating Opioid Use Treatment Through Medical Management With Infection Treatment (COMMIT) trial was a multisite randomized clinical trial with enrollment from August 19, 2020, through October 31, 2023, at 3 US hospital systems in Connecticut, Pennsylvania, and South Carolina. Eligible participants were individuals hospitalized with a diagnosis of moderate to severe OUD according to the *Diagnostic and Statistical Manual of Mental Disorders* (Fifth Edition) and concurrent infection. Intent-to-treat outcomes were assessed at the end of the 12-week intervention period.

**Interventions:**

Participants were randomized 1:1 to receive ID-LAB or TAU during treatment for infection in a hospital setting or early after discharge. All participants received a nurse care medical management intervention.

**Main Outcomes and Measures:**

The primary outcome was the proportion of patients who received any form of MOUD at 12 weeks after randomization. Models were adjusted by site, prescription of MOUD in the 30 days prior to hospitalization, and the baseline value of each outcome when assessable.

**Results:**

Of the 171 participants who were enrolled, 86 were randomized to the ID-LAB arm and 85 to the TAU arm. A total of 88 participants (51.5%) were men, and median age was 39 (IQR, 33-47) years. At 12 weeks, there was no statistically significant difference in receipt of MOUD between the ID-LAB and TAU groups, with 51 patients (59.3%) and 46 (54.1%), respectively, receiving MOUD (adjusted rate ratio, 1.01; 95% CI, 0.78-1.30).

**Conclusions and Relevance:**

In this randomized clinical trial comparing initiation of LAB for OUD with ID management in the hospital setting compared with TAU, there was no difference between arms in the receipt of MOUD at 12 weeks. The TAU arm had higher retention than anticipated. These findings suggest that hospitalization with an infection related to drug use may present an opportunity to identify OUD and initiate MOUD that may include injectable LAB. The nurse case management services provided to all participants should be evaluated in future studies.

**Trial Registration:**

ClinicalTrials.gov Identifier: NCT04180020

## Key Points

**Question:**

Does initiating long-acting buprenorphine (LAB) with infectious disease (ID) management (ID-LAB) for hospitalized persons with opioid use disorder (OUD) and infection improve receipt of medications for OUD (MOUD) 12 weeks after randomization?

**Findings:**

In this randomized clinical trial of 171 adults with infections and OUD, there was no difference in the proportion who received MOUD at 12 weeks between the ID-LAB (59%) and treatment as usual (54%) arms.

**Meaning:**

These findings suggest that patient preference and shared decision-making should guide which formulation of MOUD is started during hospitalization for infections in patients with OUD.

## Teaser

This randomized clinical trial of patients with opioid use disorder (OUD) hospitalized for infection examines whether initiating long-acting buprenorphine plus infectious disease management vs treatment as usual improves receipt of medications for OUD at 12 weeks.

## Introduction

Substance use–related overdoses in the US have claimed over 1 million lives since 1999, with numbers only recently beginning to decline after peaking at over 110 000 deaths per year.^1^ Opioids are the leading contributor, involved in over 75% of all overdose deaths, largely driven by illicitly manufactured fentanyl. The ongoing substance use epidemic is also associated with increases in related severe infections, such as infective endocarditis and acute hepatitis C virus (HCV) infection.^2,3^ Hospitalizations due to concurrent opioid use disorder (OUD) and infections are rising, and while the conditions are often related, they tend to be managed by separate inpatient teams, highlighting the siloing of hospitalists and addiction medicine, addiction psychiatry, and infectious diseases (ID) specialists in health care delivery.^4^

Medications for opioid use disorder (MOUD; ie, buprenorphine, methadone, and extended-release naltrexone) reduce opioid craving, return to opioid use, overdose, and death. However, in 2021, of the 2.5 million adults in the US with OUD, only 22% received MOUD.^5,6,7,8^

Novel approaches are needed to expand access to MOUD, particularly for institutions that lack addiction specialists. Integration of OUD and ID treatment is well supported in the literature,^9,10,11,12^ and ID clinicians have a unique opportunity to help patients understand the impact of OUD and infection, initiate MOUD, and participate in outpatient follow-up, ensuring continuity of care for both infections and OUD.^13^

Monthly injectable long-acting buprenorphine (LAB; Sublocade) was approved by the US Food and Drug Administration (FDA) in 2017 and is effective in treating OUD,^14^ though adoption of this treatment has been limited largely to the outpatient setting. LAB is an effective treatment option for patients who have difficulty adhering to daily sublingual buprenorphine or methadone.

This study evaluated LAB combined with ID management (ID-LAB) compared with treatment as usual (TAU), with nurse care manager (NCM) support in both arms, for persons with OUD hospitalized with 1 or more infections who were interested in buprenorphine treatment. MOUD receipt at 12 weeks after randomization was examined as the primary outcome, and ID treatment and substance use–related outcomes were secondary end points. We hypothesized that ID-LAB would improve receipt of MOUD and ID outcomes compared with TAU.

## Methods

### Study Design

The Coordinating Opioid Use Treatment Through Medical Management With Infection Treatment (COMMIT) trial was a prospective 12-week, multisite randomized clinical trial of the effectiveness of ID-LAB vs TAU (NCT04180020).^15^ The trial protocol is provided in Supplement 1. Protocol details have been previously published^15^; the study conception and design were based on previous trials showing efficacy in ID and addiction outcomes using integrated care models with depot medication formulations for OUD.^9,10,11,12^ Participants were enrolled in US medical hospital settings that serve mixed urban, suburban, and rural populations across 3 US states: Yale New Haven Hospital in New Haven, Connecticut; Prisma Health System in Greenville, South Carolina; and Penn State Milton S. Hershey Medical Center in Hershey, Pennsylvania. Participants in the study were representative of the hospitalized populations in those settings. Enrollment at these locations occurred from August 19, 2020, through October 31, 2023. The Medical University of South Carolina institutional review board (IRB) served as the single IRB and approved the study. This study adhered to the Consolidated Standards of Reporting Trials (CONSORT) 2010 statement guidelines. Written informed consent was obtained from all participants, and study visits were compensated with a cash value up to $290.

### Participants

Eligible participants were aged 18 years or older, diagnosed with moderate to severe OUD according to the *Diagnostic and Statistical Manual of Mental Disorders* (Fifth Edition) (*DSM-5*),^16^ interested in MOUD treatment with buprenorphine, hospitalized with a known or suspected infection (including uncontrolled HIV infection, hepatitis B, or HCV infection with a detectable viral load), willing to accept assignment to either the ID-LAB or TAU arm, and willing to participate in research follow-up visits. Exclusion criteria included a severe medical or psychiatric disability making participation unsafe; pregnancy, planning conception, or breastfeeding; medical contraindication to buprenorphine; moderate-severe liver impairment; durable maintenance with MOUD for 30 days prior to hospitalization and intending to continue that MOUD after discharge; or inability or unwillingness to provide informed consent.

### Intervention Groups

Eligible participants were recruited while hospitalized and randomized 1:1 to 1 of 2 study arms: (1) comanagement of OUD with LAB integrated into their ID care (ID-LAB) or (2) TAU. Randomization was done using randomly permuted blocks of 4 stratified by study site through a centralized computer system. Study arms were unblinded and had no placebo injections.

#### ID-LAB

OUD was comanaged by ID services, hospitalist teams, and addiction medicine and psychiatry services if available. Details of LAB administration protocols have been previously published.^15^ The timing of the initial LAB injection took into consideration anticipated medical treatment, including need for surgery or pain management, and was sometimes completed in the outpatient follow-up period. Prior to the injection, a minimum of 16 mg of sublingual buprenorphine had to be administered for 2 days. An FDA Investigational New Drug application was obtained given the shorter buprenorphine induction period of 2 days compared with the standard of 7 days at that time for the original FDA-approved administration during the study course. Participants were observed for 2 hours after administration, with assessments for precipitated withdrawal and oversedation performed using the Clinical Opiate Withdrawal Scale (COWS)^17^ and work by Ramsay et al.^18^ A total of 3 LAB injections (300 mg) were offered every 28 days, with a dosing window of 2 days before or 14 days after the scheduled injection date.

#### TAU

TAU reflected usual care for management of OUD at the participating hospitals. OUD was typically managed by hospitalists, though most sites had deployed addiction medicine consultation services oriented toward initiating MOUD before or during the study.

#### NCM Model

All participants regardless of study arm received counseling based on the NCM model,^19,20^ which used nurses and advanced practice practitioners to follow up with participants throughout their study participation and provided the standardized medical management counseling,^21^ a brief 15-minute intervention that promotes recovery, adherence to MOUD, and all aspects of the OUD and ID treatment plan. The NCM communicated with the participant twice a week during hospitalization and weekly for the remainder of the study and facilitated linkage to outpatient substance use treatment after the study. All participants received education about OUD, formulations of MOUD, and naloxone distribution and other harm reduction education.^15^

### Study End Points

The primary outcome was a binary indicator defined as receipt of any form of MOUD as indicated in the electronic medical record (EMR), by confirmation from a community treatment program, and/or in the electronic prescription drug monitoring program (PDMP) at 12 weeks after randomization.^15^ As reported in the published protocol,^15^ participants were considered to have received MOUD at week 12 if they were taking oral MOUD (sublingual buprenorphine, methadone) and received their last documented MOUD dose (obtained from the EMR or, if not available, through the PDMP and for methadone via confirming from the methadone programs) within 14 days of the week 12 follow-up visit and assessment. Participants receiving depot formulations (LAB, extended-release naltrexone) were considered to have received MOUD if their last documented dose occurred within 42 days (28 days plus a 14-day window) of the week 12 follow-up visit and assessment.

Secondary outcomes included ID outcomes: completion of antimicrobial treatment (binary), defined as the completion of prescribed antimicrobial therapy without missed doses for the index infection, and cure of the index infection. Other secondary outcomes were opioid use outcomes (days of opioid use obtained by urine toxicology screening results and self-report through Timeline Followback [TLFB]^22^), quality of life (QOL), psychiatric disorder symptoms (posttraumatic stress disorder [PTSD], depression, or attention-deficit/hyperactivity disorder [ADHD]), pain, HIV risk behaviors, and adverse events.

### Assessment

Study assessments occurred at baseline and weeks 4, 8, 12 (end of intervention), and 24.^15^ Urine toxicology screening was performed using the 13-panel SAFElife T-Cup multidrug urine test, and rapid point of care HIV and HCV testing was done with reflex positive viral load testing if data were not available in the EMR. At baseline, self-reported demographic information was collected, including sex at birth, current gender identity (cisgender man, cisgender woman, transgender man, and transgender woman), race (Asian, Black or African American, American Indian or Alaska Native, Pacific Islander, White, multiracial, and other or unknown [included people who identified as Hispanic ethnicity but did not self-identify as any race or who did not answer]), ethnicity (Hispanic or non-Hispanic), housing status, educational level, and marital status. Race and ethnicity were included in the analysis to describe the study population and as potential factors associated with the receipt of MOUD. The Mini-International Neuropsychiatric Interview, *DSM-5*, version 7.0.2,^23^ was used to establish diagnoses of current moderate to severe substance use disorder and major psychiatric disorders. Scheduled visit assessments included urine toxicology screening and pregnancy testing, self-report of drug use by TLFB, a 10-point visual analog scale for opioid craving, depressive symptoms using the Patient Health Questionnaire–9,^24,25^ PTSD symptoms using the Posttraumatic Stress Disorder Checklist for *DSM-5*,^26^ ADHD symptoms using the Adult ADHD Self Report Scale,^27^ QOL using the World Health Organization Quality of Life Brief Version,^28^ the Alcohol Use Disorders Identification Test for hazardous drinking,^29^ pain using the Modified Pain, Enjoyment of Life, and General Activity Scale (PEG) adapted from the PEG Pain Scale,^30^ HIV risk behaviors (sexual and injection drug use) using the HIV Risk Behavior Tool,^31^ and adverse event assessment.

### Statistical Analysis

All analyses were performed based on an intent-to-treat sample at a 2-sided significance level of *P* <.05. A sample size of 200 participants was chosen to detect a clinically meaningful difference of at least 19.7% on the primary outcome with a type I error rate of 0.05 and a power of 80%. One-month postdischarge MOUD receipt rates ranged from 45% to 70% in published literature.^32,33^ We estimated a 12-week MOUD receipt rate of 60% in the ID-LAB arm and 40% in the TAU arm. The effect of randomization to the ID-LAB arm compared with the TAU arm was estimated with generalized linear models with appropriate link function (identity link function for continuous outcomes following normal distributions or log link function for binary outcomes). When models failed to converge, logistic regression was used to estimate the risk ratio (RR) and assess the effect of randomization (eTables 1 and 2 in Supplement 2 provide model parameters).^34^ Models were adjusted by site, prescription of MOUD in the 30 days prior to hospitalization, and the baseline value of each outcome (when baseline could be assessed). The RR (for dichotomous variables) or adjusted mean difference (for continuous variables) and their 95% CI estimated the treatment effect. The primary outcome was the binary outcome of active MOUD receipt (yes, no), modeled as a function of treatment condition (ID-LAB vs TAU).

For the primary outcome and relevant secondary outcomes, participants lost to follow-up were considered to have not continued treatment with MOUD or a prescribed antimicrobial course. Other missing outcome data for patients lost to follow-up were treated as random except for substance use–related outcomes, for which recurrence of opioid or other substance use was assumed.^35,36^

To assess the differences in adverse events, χ^2^ analyses were performed for comparisons with values of 6 observations in each group. Fisher exact analyses were conducted for comparisons with 5 or fewer observations. Data were analyzed with SAS, version 9.4 (SAS Institute Inc).

## Results

### Participants

**Figure 1.**
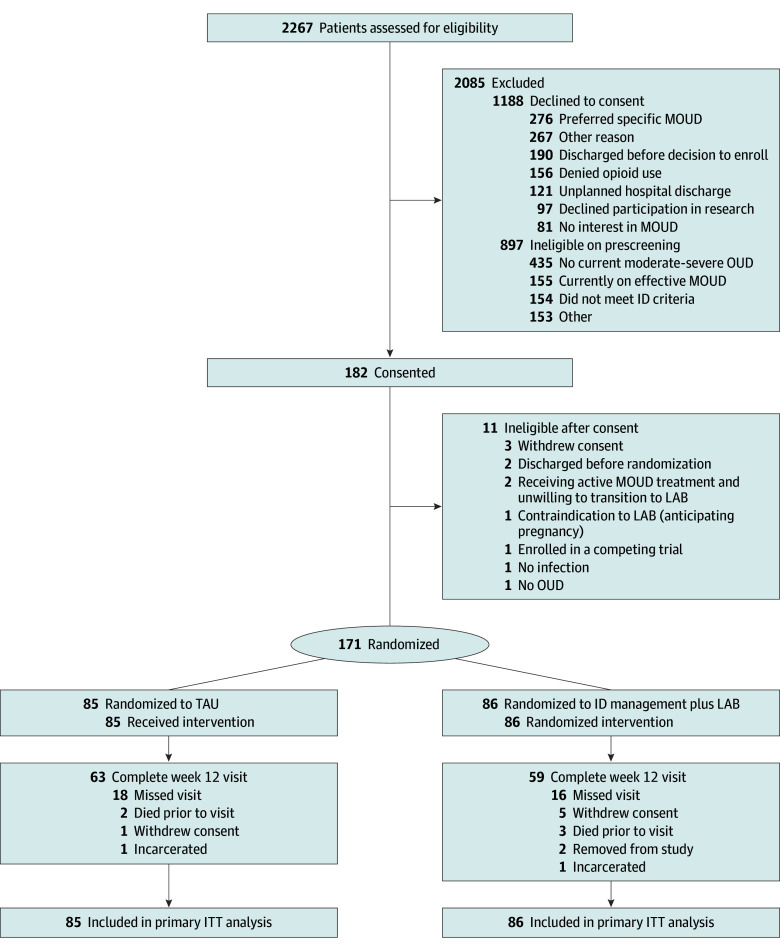
CONSORT Diagram ID indicates infectious disease; ITT, intent to treat; LAB, long-acting buprenorphine; MOUD, medications for opioid use disorder; OUD, opioid use disorder; TAU, treatment as usual.

**Figure 2.**
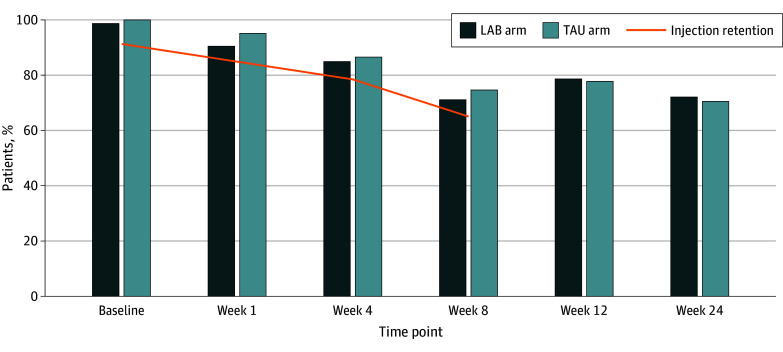
Interview and Study Medication Injection Retention Details on interview retention are given in eTable 3 in Supplement 2.

Of 2267 patients assessed for eligibility, 171 were eligible, enrolled, and randomized (86 to ID-LAB and 85 to TAU) (Figure 1). The demographic and clinical characteristics of the 171 randomized participants are shown in Table 1. Eighty-eight participants (51.5%) identified as cisgender men, 82 (47.9%) as cisgender women, none as transgender men, and 1 (0.6%) as a transgender woman, and median age was 39 (IQR, 33-47) years. Three participants (1.8%) identified as American Indian or Alaska Native, none identified as Asian, 14 (8.2%) identified as Black or African American, 137 (80.1%) identified as White, 7 (4.1%) identified as multiracial, and 10 (5.8%) reported other or unknown race. Eighteen (10.5%) identified as Hispanic and 153 (89.5%) as non-Hispanic. The study population was predominantly unhoused or unstably housed (109 of 170 [64.1%]) and had annual income less than $25 000 (100 of 165 [60.6%]). About half (96 of 170 [56.5%]) had health insurance, mainly Medicaid. The most frequently reported illicit opioid used was heroin (117 of 171 participants [68.4%]) followed by fentanyl (76 of 169 [45.0%]). Thirty-four participants (19.9%) had been prescribed a form of MOUD in the 30 days prior to hospitalization. Participants had high rates of moderate to severe depressive symptoms (117 of 166 [70.5%]). Interview and LAB injection retention through the study duration are shown in Figure 2 and eTable 3 in Supplement 2. Of the 86 participants randomized to receive ID-LAB, 4 (4.7%) withdrew consent prior to injection, 1 (1.2%) declined the injection, and 1 (1.2%) died. Of the remaining 80 participants, 73 (91.2%) received the first injection. Reasons for not receiving the first injection included self-discharge and loss to follow-up; clinical contraindications, such as abdominal wall wounds restricting the ability to perform abdominal subcutaneous LAB injections; and concurrent medication interactions. Of the 73 participants that received a first injection, 39 (53.4%) received an injection 1 day prior to discharge, 18 (24.7%) on the day of hospital discharge, and 16 (21.9%) after discharge. Fifteen participants (17.4%) received a 100-mg dose of LAB for their third dose, as per provider discretion. In the TAU arm, 80 of 85 participants (94.1%) received methadone or buprenorphine during hospitalization.

**Table 1.** Baseline Participant Characteristics

Characteristic	Participants^a^		
	Overall (N = 171)	ID-LAB (n = 86)	TAU (n = 85)
**Demographic characteristics**			
Study site			
South Carolina	107 (62.6)	53 (61.6)	54 (63.5)
Connecticut	55 (32.2)	28 (32.6)	27 (31.8)
Pennsylvania	9 (5.3)	5 (5.8)	4 (4.7)
Age, median (IQR), y	39 (33-47)	38 (32-46)	40 (33-47)
Gender			
Cisgender man	88 (51.5)	46 (53.5)	42 (49.4)
Cisgender woman	82 (47.9)	39 (45.3)	43 (50.6)
Transgender man	0	0	0
Transgender woman	1 (0.6)	1 (1.2)	0
Ethnicity			
Hispanic	18 (10.5)	4 (4.7)	14 (16.5)
Non-Hispanic	153 (89.5)	82 (95.3)	71 (83.5)
Race			
American Indian or Alaska Native	3 (1.8)	1 (1.2)	2 (2.4)
Asian	0	0	0
Black or African American	14 (8.2)	5 (5.8)	9 (10.6)
White	137 (80.1)	74 (86.0)	63 (74.1)
Multiracial	7 (4.1)	4 (4.7)	3 (3.5)
Other or unknown^b^	10 (5.8)	2 (2.3)	8 (9.4)
Housing status			
Unhoused	42/170 (24.7)	21/85 (24.7)	21/85 (24.7)
Unstable housing	67/170 (39.4)	30/85 (35.3)	37/85 (43.5)
Stable housing	61/170 (35.9)	34/85 (40.0)	27/85 (31.8)
Educational level ≥ high school	135/169 (79.9)	67/84 (79.8)	68/85 (80.0)
Marital status			
Living with partner	17/170 (10.0)	12/85 (14.1)	5/85 (5.9)
Married	21/170 (12.4)	11/85 (12.9)	10/85 (11.8)
Divorced	39/170 (22.9)	21/85 (24.7)	18/85 (21.1)
Never married	69/170 (40.6)	29/85 (34.1)	40/85 (47.1)
Separated	18/170 (10.6)	8/85 (9.4)	10/85 (11.8)
Widowed	6/170 (3.5)	4/85 (4.7)	2/85 (2.4)
Income group			
<$5000	52/165 (31.5)	25/81 (30.8)	27/84 (32.1)
$5000-$9999	12/165 (7.3)	6/81 (7.4)	6/84 (7.1)
$10 000-$24 999	36/165 (21.8)	16/81 (19.8)	20/84 (23.8)
$25 000-$49 999	33/165 (20.0)	17/81 (21.0)	16/84 (19.0)
≥$50 000	32/165 (11.5)	17/81 (21.0)	15/84 (17.9)
Type of insurance			
Medicaid	76/96 (79.2)	38/50 (76.0)	38/46 (80.9)
Medicare	3/96 (3.1)	2/50 (4.0)	1/46 (2.1)
Private	13/96 (13.5)	7/50 (14.0)	6/46 (12.8)
Other	4/96 (4.2)	3/50 (6.0)	1/46 (2.1)
HIV risk behaviors 30 d prior to enrollment			
Engaged in condomless sex	71/165 (43.3)	35/81 (43.2)	36/84 (42.9)
Condomless sex partners, median (IQR), No. (n = 71)	1 (1-1)	1 (1-2)	1 (1-1)
Engaged in injection drug use	117/169 (69.2)	64/85 (75.3)	53/84 (63.1)
Shared injection drug equipment	39/116 (33.6)	21/63 (33.3)	18/53 (34.0)
**Psychological characteristics**			
Quality of life, WHOQOL-BREF score, median (IQR)^c^			
Physical health (n = 169)	39.3 (25.5-60.7)	39.3 (21.4-57.1)	42.9 (28.6-60.7)
Psychological (n = 170)	45.8 (37.5-62.5)	45.8 (37.5-66.7)	45.8 (37.5-62.5)
Social relationships (n = 168)	50.0 (29.2-75.0)	54.2 (29.2-75.0)	45.8 (29.2-66.7)
Environmental (n = 170)	56.3 (40.6-71.9)	56.7 (40.6-78.1)	53.1 (40.6-68.8)
Provisional PTSD diagnosis, PTSD-PCL	88/170 (51.8)	42/85 (49.4)	46/85 (54.1)
Depression severity, PHQ-9			
None or mild	49/166 (29.5)	23/83 (27.8)	26/83 (31.3)
Moderate or greater	117/166 (70.5)	60/83 (72.3)	57/83 (68.7)
ADHD provisional diagnosis, ASRS	112 (65.5)	62 (72.1)	50 (58.8)
Pain score, PEG, median (IQR) (n = 170)^d^	6.0 (3.2-7.8)	6.0 (4.0-7.0)	6.0 (3.0-8.0)
**Substance use–related characteristics**			
Opioid Craving Scale score, median (IQR) (n = 166)^e^	1.00 (1.00-5.00)	1.00 (1.00-5.00)	1.00 (1.00-5.00)
Mild or greater opioid withdrawal, COWS	27/170 (15.9)	8/85 (9.4)	19/85 (22.4)
Hazardous or harmful drinking, AUDIT	38 (22.2)	15 (17.4)	23 (27.1)
Co-occurring *DSM-5* stimulant use disorder, MINI	92 (53.8)	44 (51.2)	48 (56.4)
Prescribed MOUD in past 30 d	34 (19.9)	19 (22.1)	15 (17.6)
Methadone	13 (7.7)	7 (8.3)	6 (7.1)
Buprenorphine	20 (11.8)	12 (14.0)	8 (9.4)
Extended-release naltrexone	1 (0.6)	0	1 (1.2)
Positive urine toxicology screening result at enrollment			
Opiates	34/166 (20.5)	23/84 (27.4)	11/82 (13.4)
Oxycodone	40/166 (24.1)	20/84 (23.8)	20/82 (24.4)
Fentanyl	79/165 (47.9)	40/84 (47.6)	39/81 (48.1)
Methadone	36/165 (21.8)	22/84 (26.2)	14/81 (17.3)
Buprenorphine	108/164 (65.9)	57/84 (67.9)	51/80 (63.8)
Cocaine	8/166 (4.8)	3/84 (3.6)	5/82 (6.1)
Methamphetamine	19/166 (11.4)	7/84 (8.3)	12/82 (14.6)
Benzodiazepine	49/166 (29.5)	29/84 (34.5)	20/82 (24.4)
Self-reported substances used 30 d before hospitalization, TLFB			
Heroin	117/171 (68.4)	59/86 (68.6)	58/85 (68.2)
Prescription opioids	38/169 (22.5)	21/85 (24.7)	17/84 (20.2)
Fentanyl	76/169 (45.0)	36/85 (42.4)	40/84 (47.6)
Other opioids	15/169 (8.9)	9/85 (10.6)	6/84 (7.1)
Cocaine	57/169 (33.7)	30/85 (35.3)	27/84 (32.1)
Methamphetamine	79/169 (46.7)	40/85 (47.1)	39/84 (46.4)
**Index infection and hospitalization characteristics**			
Visited HCP in past 12 mo excluding urgent care	62/170 (36.5)	28/85 (32.9)	34/85 (40.0)
Covered by health insurance 30 d before interview	96/170 (56.5)	50/85 (58.8)	46/85 (54.1)
Hospitalization duration, median (IQR), d (n = 170)	14 (6-30)	14 (6-30)	14 (7-28)
Unplanned hospital discharge	24/169 (14.1)	14/84 (16.7)	10/85 (11.8)
Index infection			
HIV			
Diagnosis	4 (2.3)	1 (1.2)	3 (3.5)
Viral load ≥200 copies/mL	1 (0.6)	1 (1.2)	0
Hepatitis C			
Antibody positive	114 (66.7)	61 (70.9)	53 (62.4)
With detectable viral load	71 (41.5)	35 (40.7)	36 (42.4)
Hepatitis B	2 (1.2)	2 (2.3)	0
Bloodstream infection	77 (45.0)	41 (47.7)	36 (42.4)
Endocarditis	35 (18.1)	20 (23.3)	15 (17.6)
Septic arthritis	28 (16.4)	14 (16.3)	14 (16.5)
Osteomyelitis	31 (18.1)	15 (17.4)	16 (18.8)
Pneumonia or respiratory infection (non–COVID-19)	33 (19.3)	14 (16.3)	19 (22.4)
Septic thrombophlebitis	4 (2.3)	2 (2.3)	2 (2.4)
Skin or skin structure infection	35 (20.5)	24 (27.9)	11 (12.9)
Abscess, including skin or soft tissue, intra-abdominal, epidural, or other	57 (33.3)	27 (31.4)	30 (35.3)
COVID-19	6 (3.5)	3 (3.5)	3 (3.5)
Sexually transmitted infection	8 (4.7)	5 (5.8)	3 (3.5)
Other	6 (3.5)	1 (1.2)	5 (5.9)

Abbreviations: ADHD, attention-deficit/hyperactivity disorder; ASRS, Adult Attention-Deficit/Hyperactivity Disorder Self-Report Scale; AUDIT, Alcohol Use Disorders Identification Test; COWS, Clinical Opiate Withdrawal Scale; *DSM-5*, *Diagnostic and Statistical Manual of Mental Disorders* (Fifth Edition); HCP, health care practitioner; ID, infectious diseases management; LAB, long-acting buprenorphine; MINI, Mini International Neuropsychiatric Interview; MOUD, medications for opioid use disorder; PEG, Pain, Enjoyment, and General Activity Scale; PHQ-9, Patient Health Questionnaire–9; PTSD, posttraumatic stress disorder; PTSD-PCL, Posttraumatic Stress Disorder Checklist; TAU, treatment as usual; TLFB, Timeline Followback; WHOQOL-BREF, World Health Organization Quality of Life Brief Version.

^a^ Data are presented as number or number/total number (percentage) of participants unless otherwise indicated.

^b^ Includes 7 people (4.1%) who identified as Hispanic but did not self-identify as any race and 3 (1.8%) who did not answer and, therefore, their race was unknown.

^c^ WHOQOL-BREF score range, 0 to 100, with higher scores indicating better quality of life.

^d^ PEG score range, 0 to 10, with higher scores indicating more pain.

^e^ Opioid Craving Scale score range, 0 to 10, with higher scores indicating more significant cravings experienced at time of interview.

### Primary Outcome

At the week 12 time point, 51 participants in the ID-LAB arm (59.3%) and 46 in the TAU arm (54.1%) were receiving MOUD (adjusted RR, 1.01; 95% CI, 0.78-1.30; *P* = .94) (Table 2). Sublingual buprenorphine formulations were the most common MOUD received in the TAU arm (37 [43.5%]); 5 patients in the TAU arm (5.9%) received LAB and 4 (4.7%) received methadone. A total of 37 in the ID-LAB arm (43.0%) and 40 in the TAU arm (47.1%) were receiving MOUD at the week 24 time point (*P* = .67).

**Table 2.** Intention-to-Treat Primary and Secondary Outcomes at 12 and 24 Weeks

Outcome^a^	12 wk				24 wk			
	ID-LAB (n = 86)^b^	TAU (n = 85)^b^	Estimate (95% CI)^c^	*P* value	ID-LAB (n = 86)^b^	TAU (n = 85)^b^	Estimate (95% CI)^c^	*P* value
Primary outcome								
Enrollment in MOUD treatment	51 (59.3)	46 (54.1)	1.01 (0.78 to 1.30)	.94^d^	37 (43.0)	40 (47.1)	0.94 (0.69 to 1.27)	.67^d^
Secondary outcomes								
Index infection, 1-time outcome,								
Treatment completed	61/77 (79.2)	67/78 (85.9)	1.53 (0.76 to 3.07)	.23^d^	NA	NA	NA	NA
Cured	65/82 (79.3)	68/84 (81.0)	0.96 (0.84 to 1.11)	.62^d^	NA	NA	NA	NA
HCV detectable viral load	38/56 (67.9)	40/59 (67.8)	0.85 (0.59 to 1.23)	.38^d^	NA	NA	NA	NA
HIV risk factor								
Condomless sex	19/59 (32.2)	25/62 (40.3)	1.08 (0.85 to 1.38)	.53^d^	17/51 (33.3)	20/55 (36.4)	0.99 (0.75 to 1.31)	.97^d^
Shared injection drug use equipment	3/59 (5.1)	2/62 (3.2)	1.34 (0.17 to 8.28)	.77^e^	1/51 (2.0)	2/55 (3.6)	2.90 (0.27 to 16.10)	.37^e^
WHOQOL-BREF score^f^^,^^g^	86.8 (1.9)	82.5 (2.0)	3.16 (−2.01 to 8.33)	.23	87.3 (1.9)	82.7 (2.2)	3.59 (−1.79 to 8.97)	.19
Pain, PEG score^h^^,^^g^	2.8 (0.4)	3.7 (0.4)	−0.78 (−1.83 to 0.16)	.10	2.8 (0.4)	3.4 (0.4)	−0.49 (−1.57 to 0.58)	.37
PTSD	NA	NA	NA	NA	16/50 (32.0)	21/54 (38.9)	1.04 (0.78 to 1.39)	.79^d^
PHQ-9 score^i^	7.59 (0.82)	8.76 (0.84)	−0.86 (−2.88 to 1.15)	.40	7.13 (0.81)	8.91 (0.87)	0.86 (1.15 to 2.88)	.40
TLFB of reported substance use, d^g^	11.3 (1.5)	12.5 (1.5)	−1.71 (−5.72 to 2.29)	.40	14.4 (1.5)	15.2 (1.6)	−1.07 (−5.29 to 3.15)	.62
Urine toxicology screening negative for opioids	31 (36.0)	39 (45.9)	1.18 (0.92 to 1.50)	.19^d^	32 (37.2)	37 (43.5)	1.17 (0.93 to 1.46)	.17^d^

Abbreviations: HCV, hepatitis C virus; ID, infectious disease; LAB, long-acting buprenorphine; MOUD, medications for opioid use disorder; NA, not applicable; PEG, Pain, Enjoyment, and General Activity Scale; PHQ-9, Patient Health Questionnaire–9; PTSD, posttraumatic stress disorder; QOL, quality of life; TAU, treatment as usual; TLFB, Timeline Followback; WHOQOL-BREF, World Health Organization Quality of Life Brief Version.

^a^ For outcomes pertaining to opioid use, participants were assumed to be not enrolled or not abstinent when data were missing.

^b^ Data are presented as number or number/total number (percentage) for dichotomous variables and mean (SE) for continuous variables.

^c^ Adjusted risk ratios are reported for dichotomous variables and adjusted mean differences for continuous variables.

^d^ Modeled using log-linked binomial regression.

^e^ Modeled using a logistic regression that did not converge, with risk ratios estimated using the method of Zhang and Yu.^34^ Site was removed as a covariate, as participants from only 1 site reported shared injection drug use equipment.

^f^ WHOQOL-BREF score range, 0 to 100, with higher scores indicating better quality of life.

^g^ Four participants (2.3%) withdrew from the study, and 1 (0.6%) died unrelated to index infection.

^h^ PEG score range, 0 to 10, with higher scores indicating more pain.

^i^ PHQ-9 score range, 0 to 27, with higher scores indicating more severe depression.

### Secondary Outcomes

#### Substance Use and Opioid Outcomes

At the week 12 time point, no difference was seen in illicit opioid positivity per urine toxicology screening between study arms (Table 2). Thirty-one of 86 samples (36.0%) were negative for illicit opioids in the ID-LAB arm compared with 39 of 85 (45.9%) in the TAU arm (*P* = .19). Participants endorsed a mean (SD) 11.3 (1.5) days of reported opioid use in the preceding 30 days in the ID-LAB arm and 12.5 (1.5) days in the TAU arm (*P* = .38). By the week 24 time point, 32 participants in the ID-LAB arm (37.2%) and 37 in the TAU arm (43.5%) reported any use of illicit opioids.

#### ID Outcomes

Participants were enrolled with various and often multiple infections (Table 1). The most common were bacteremia (73 participants [42.6%]), viremic HCV infection (71 [41.5%]), abscesses (57 [33.3%]), skin and soft tissue infections (34 [19.8%]), and infectious endocarditis (31 [18.1%]). As shown in Table 2, the treatment outlined for the index infection was completed in 61 of 77 participants in the ID-LAB arm (79.2%) and 67 of 78 in the TAU arm (85.9%) (*P* = .23). Data were not applicable for 16 patients (9.4%) due to death or no need for antimicrobial treatment. Additionally, there was no difference in infection cure rates between arms, with 65 of 82 in the ID-LAB arm (79.3%) and 68 of 84 in the TAU arm (81.0%) determined to be clinically cured at the 12-week time point.

#### Safety Outcomes

A total of 135 participants (78.9%; 67 in the ID-LAB arm [77.9%] and 68 in the TAU arm [80.0%]) experienced an adverse event (AE), with no statistically significant difference between the groups (*P* = .97). The most common AEs were opioid withdrawal, skin or soft tissue infection, arthralgias, nausea, and rash (Table 3). Furthermore, 70 participants (40.9%; 35 in the ID-LAB arm [40.7%] and 35 in the TAU arm [41.2%]; *P* = .95) experienced a nonfatal serious AE. Of the 127 total serious AEs, 3 (2.4%) were possibly study related, while 55 (43.3%) were related to the index infection. Injection site reactions occurred in 9 patients in the ID-LAB arm (10.5%), and all were mild to moderate. There were no cases of LAB-induced precipitated withdrawal. One patient (1.2%) discontinued LAB due to the development of a medical contraindication. There were 15 reported nonfatal overdoses, 4 of which (26.7%) occurred in the ID-LAB arm and 11 (73.3%) in the TAU arm (*P* = .04). There were 8 deaths (4 [50.0%] in each arm), all unrelated to the study intervention, and none were due to overdose. Causes of death for those in the ID-LAB arm were relapse of index infection, acute respiratory failure, and severe bilateral pulmonary emboli with right ventricular dysfunction, and the cause of 1 death was undetermined. Causes of death in the TAU arm were sepsis, S*taphylococcus aureus* bacteremia, and gastrointestinal hemorrhage, and 2 had undetermined cause.

**Table 3.** Summary of Study-Emergent Adverse Events

	Frequency, No. (%)^a^					
	Overall		TAU		ID-LAB	
	Events	Participants	Events	Participants	Events	Participants
**AEs and SAEs**						
Total AEs, No.	554	135	256	68	298	67
Total SAEs, No.	127	70	55	35	72	35
Opioid withdrawal						
AEs	33 (6.0)	23 (17.0)	18 (7.0)	9 (13.2)	15 (5.0)	14 (20.9)
SAEs	1 (0.8)	1 (1.4)	1 (1.8)	1 (2.9)	0	0
Skin or soft tissue infection						
AEs	22 (4.0)	16 (11.9)	14 (5.5)	9 (13.2)	8 (2.7)	7 (10.4)
SAEs	45 (35.4)	30 (42.9)	20 (36.4)	14 (40.0)	25 (29.1)	16 (45.7)
Arthralgia						
AEs	17 (3.1)	14 (10.4)	8 (3.1)	6 (8.8)	9 (3.0)	8 (11.9)
SAEs	1 (0.8)	1 (1.4)	0	0	1 (1.4)	1 (2.9)
Nausea						
AEs	26 (4.7)	15 (11.1)	6 (2.3)	5 (7.4)	11 (3.7)	10 (14.9)
SAEs	0	0	0	0	0	0
Rash						
AEs	14 (2.5)	14 (10.4)	7 (2.7)	7 (10.3)	7 (2.3)	7 (10.4)
SAEs	0	0	0	0	0	0
Headache						
AEs	17 (3.1)	13 (9.6)	4 (1.6)	4 (5.6)	13 (4.4)	9 (13.4)
SAEs	0	0	0	0	0	0
Edema						
AEs	12 (2.2)	11 (8.1)	7 (2.7)	6 (8.8)	5 (1.7)	5 (7.5)
SAEs	0	0	0	0	0	0
Abdominal pain						
AEs	12 (2.2)	11 (8.1)	6 (2.3)	6 (8.8)	6 (2.0)	5 (7.5)
SAEs	5 (3.9)	2 (2.9)	1 (1.8)	1 (2.9)	4 (5.6)	1 (2.9)
Myalgia						
AEs	11 (2.0)	10 (7.4)	5 (2.0)	4 (5.6)	6 (2.0)	6 (9.0)
SAEs	0	0	0	0	0	0
Injection site reaction						
AEs	11 (2.0)	9 (6.7)^b^	0	0	11 (3.7)	9 (13.4)
SAEs	0	0	0	0	0	0
Vomiting						
AEs	8 (1.4)	8 (5.9)	4 (1.6)	4 (5.6)	4 (1.3)	4 (6.0)
SAEs	0	0	0	0	0	0
Dyspnea						
AEs	9 (1.6)	8 (5.9)	5 (2.0)	4 (5.6)	4 (1.3)	4 (6.0)
SAEs	1 (0.8)	1 (1.4)	0	0	1 (1.4)	1 (2.9)
Constipation						
AEs	10 (1.8)	7 (5.2)^c^	1 (0.4)	1 (1.5)	9 (3.0)	7 (10.4)
SAEs	0	0	0	0	0	0
Diaphoresis						
AEs	7 (1.3)	7 (5.2)	3 (1.2)	3 (4.4)	4 (1.3)	4 (6.0)
SAEs	0	0	0	0	0	0
Fatigue						
AEs	7 (1.3)	7 (5.2)	5 (2.0)	5 (7.4)	2 (0.7)	2 (3.0)
SAEs	0	0	0	0	0	0
Fever						
AEs	7 (1.3)	7 (5.2)	3 (1.2)	3 (4.4)	4 (1.3)	4 (6.0)
SAEs	0 (0.0)	0 (0.0)	0	0	0	0
Nonfatal opioid overdose						
AEs	9 (1.6)^c^	7 (5.2)	8 (3.1)	6 (8.8)	1 (0.3)	1 (1.5)
SAEs	6 (4.7)	4 (5.7)	2 (3.6)	2 (5.7)	4 (5.7)	2 (5.7)
Insomnia						
AEs	7 (1.3)	6 (4.4)	2 (0.8)	2 (2.9)	5 (1.7)	4 (6.0)
SAEs	0	0	0	0	0	0
Anemia						
AEs	6 (1.1)^c^	5 (3.7)^c^	0	0	6 (2.0)	5 (7.5)
SAEs	3 (2.4)	3 (4.3)	0	0	3 (4.2)	3 (8.6)
Back pain						
AEs	9 (1.6)^b^	4 (3.0)^c^	9 (3.5)	4 (5.6)	0	0
SAEs	0	0	0	0	0	0
Respiratory failure						
AEs	0	0	0	0	0	0
SAEs	5 (3.9)	5 (7.1)	1 (1.8)	1 (2.9)	4 (5.6)	4 (11.4)
Respiratory infection (not including COVID-19)						
AEs	5 (0.9)	4 (3.0)	0	0	5 (1.7)	4 (6.0)
SAEs	4 (3.1)	4 (5.7)	1 (1.8)	1 (2.9)	3 (4.2)	3 (8.6)
**SAE category**						
Grade^d^						
1	12 (9.4)	12 (17.1)	5 (9.1)	5 (14.3)	7 (9.7)	7 (20.0)
2	19 (15.0)	18 (25.7)	8 (14.5)	8 (22.9)	11 (15.3)	10 (26.6)
3	60 (47.2)	41 (58.6)	29 (52.7)	20 (57.1)	31 (43.1)	21 (60.0)
4	28 (22.0)	19 (56.6)	9 (16.4)	6 (17.1)	19 (26.4)	13 (37.1)
5	8 (6.3)	8 (27.1)	4 (7.3)	4 (11.4)	4 (5.6)	4 (11.3)
Resulted in rehospitalization	105 (82.7)	62 (88.6)^b^	47 (85.5)	31 (88.6)	58 (80.6)	31 (88.6)
Resulted in prolonged hospitalization	8 (6.3)	8 (11.4)	3 (5.5)	3 (8.6)	5 (6.9)	5 (14.3)
Related to study	3 (2.4)	3 (4.3)	0	0	3 (4.2)	3 (8.6)
Related to index infection	55 (43.3)	30 (42.9)	24 (43.6)	14 (40.0)	31 (43.1)	16 (45.7)

Abbreviations: AE, adverse event; ID, infectious disease; LAB, long-acting buprenorphine; SAE, serious adverse event; TAU, treatment as usual.

^a^ Where cell frequencies were less than 5, differences were tested using Fisher exact test; otherwise, χ^2^ test was used.

^b^ *P* < .01.

^c^ *P* < .05.

^d^ Grade 1, mild; 2, moderate; 3 severe; 4, life-threatening; 5, fatal.

## Discussion

To our knowledge, this is the first randomized clinical trial to test the initiation of LAB compared with TAU in persons with OUD hospitalized with infections to assess MOUD receipt in the postdischarge period. The primary outcome of receipt of any MOUD formulation at week 12, a binary variable meant to be pragmatic and clinically relevant, showed no statistically significant difference between the ID-LAB arm (51 [59.3%]) and TAU arm (46 [54.1%]; adjusted RR, 1.01; 95% CI, 0.78-1.30; *P* = .94). Of note, MOUD receipt was considerably more favorable than expected in the TAU arm based on prior studies after a 1-month discharge period^32,33^; furthermore, our findings also reflected higher rates of 3-month posthospitalization receipt of MOUD than the 20% to 40% that have been recently reported.^37,38,39,40^ This result may have been due in part to the intensive nurse case management services and the frequent contact with the research team occurring in both study arms. The case management care based on the NCM model^41^ has been shown to be successful in the implementation of buprenorphine in office-based settings.^19,20^ The NCM role in this study included weekly standardized medical management substance use counseling and may have been particularly efficacious in the vulnerable posthospital discharge period. Social determinants of health, including lack of transportation, communication (functioning telephone), and health system navigation, are common barriers faced in postacute care by persons with substance use disorders with a background of psychosocial factors and stressors, such as housing instability, stigma, and comorbid conditions. Yet, few hospital settings provide assistance with services that are essential to successful linkage to care in the postdischarge period. Of the study cohort, 64.1% reported unstable housing, highlighting the elevated level of need of this patient population. As part of this study, participants who needed cell phones were given one to maintain contact with the research team, and transportation was provided to research and clinical visits. Also, the NCM helped participants optimally access existing support for housing and food security. Quality and duration of longitudinal inpatient to outpatient support should be an area of future study as a care intervention in the management of OUD with infections.

LAB formulations have the intrinsic advantage of providing sustained therapeutic medication levels independent of the practicalities of daily sublingual dosing.^14,42,43^ The present study suggests that LAB is well tolerated in patients with OUD hospitalized with infections,^44^ with a similar adverse effect profile compared with sublingual buprenorphine or methadone as the MOUD most often received in the TAU arm; however, LAB was not superior in terms of MOUD receipt or opioid use and ID outcomes. Future moderator analyses should examine patient characteristics, such as social determinants of health, that might predict better outcomes with LAB. In this study, all sites started inpatient addiction medicine services either before or during the trial, which could have had an impact on the better-than-predicted TAU receipt of MOUD.

Participants had a high level of medical severity and comorbidity, with 42.6% having bacteremia in their index hospitalization and 41.5% having viremic HCV infection. However, this study demonstrated remarkably high completion of antimicrobial regimens and high treatment success. This parallels the effect of similar ID-OUD case management models, in which 90% treatment completion was found compared with 60% in historical controls.^9^ This impact was also in part due to a pragmatic implementation of the antimicrobial treatment plan, in which clinician-initiated treatment amendments toward second-line regimens were accommodated. These treatment regimens (eg, long-acting glycopeptides and oral antibiotics) are supported by newer clinical outcome data^45,46^ and allow for person-centered treatment with harm reduction even in premature hospital discharge.

Even though there was no statistically significant difference in the intention-to-treat primary outcome between arms in this study, LAB formulations may still confer added benefit in clinical scenarios in which TAU is less robust. For instance, it is still common for community and rural hospitals to lack multidisciplinary care teams or addiction medicine consultation services, and LAB implementation prior to hospital discharge should be further studied in these conditions. Furthermore, new FDA labeling allowing for the administration of LAB on the same day as a single dose of sublingual buprenorphine and updated injection sites (thigh, buttocks, and back of upper arm) make inpatient LAB more feasible in hospital settings.^47^ The potential superiority of long-acting treatment formulations for OUD-related infections has not been studied to our knowledge, but trial protocols for this urgent question have been proposed.^48^

### Strengths and Limitations

A strength of this randomized clinical trial is that it was, to our knowledge, the first of its kind to assess a model of care for the understudied yet common clinical scenario of infection-related hospitalization for persons with OUD. The primary outcome (receipt of MOUD at 12 weeks as a binary variable) and major secondary outcomes, such as ID antimicrobial treatment completion, were pragmatic and clinically relevant to maximize external validity. In addition, the 300-mg dose of LAB was used for all study-administered LAB to obviate questions of dose effectiveness, although site principal investigators were given the discretion to use a 100-mg dose for the third injection if there were potential concomitant medication interactions; only 15 participants (17.4%) received this dose. Another strength was the relatively high rates of retention overall in study follow-up and of retention of LAB treatment.

Limitations include that the study, with 171 participants, came close to but did not achieve the prespecified sample size of 200 participants, impacting study power and possibly obscuring an intervention effect. Goal enrollment was not reached in part due to the COVID-19 pandemic, which resulted in a complete cessation of nonessential patient contact and most research for several months. Our research operations had to evolve to include virtual contact among other processes to continue enrollment^15^ and address what is now known to have been a time of even higher opioid-related overdose deaths.^49^ The TAU condition ended up containing beneficial features not available or fully anticipated when the trial was designed, including availability of addiction medicine consultation, increasing knowledge of MOUD among the medical teams, and the NCM model. The resulting high degree of use of the opioid agonist form of MOUD for acute withdrawal management, initiation of MOUD, and linkage to care at discharge, combined with the NCM support across inpatient and subsequent outpatient care, likely represent a standard of addiction care that is not often present outside the tertiary inpatient setting, especially in community and rural hospitals, and warrant future study.

## Conclusions

In this randomized clinical trial of hospitalized patients with infections and OUD, LAB prior to discharge was not superior to TAU in MOUD receipt at 12 weeks. Both groups had equivalent receipt of MOUD and antimicrobial treatment completion. The TAU arm had a higher rate of postdischarge MOUD receipt than expected, possibly due to the care and attention provided through the NCM model, follow-up by the research teams, and increased availability of addiction medicine consultation services. Future research is needed to evaluate the use of intensive NCM services and longer-acting formulations of MOUD to improve outcomes for both addiction and infection while improving longer-term MOUD retention in this population.

## Notes

Supplement 1.
Trial Protocol

Supplement 2. **eTable 1.** Week 12 Outcomes **eTable 2.** Week 24 Outcomes **eTable 3.** Interview Retention

Supplement 3.
Data Sharing Statement
