# Supplementary material for: Initiating Injectable Buprenorphine in People Hospitalized With Infections: A Randomized Clinical Trial
Source: JAMA Netw Open. 2025 May 30;8(5):e2513000. doi: 10.1001/jamanetworkopen.2025.13000 (PMC12125644; doi:10.1001/jamanetworkopen.2025.13000)
Supplement: Supplement 1. — Trial Protocol [file jamanetwopen-e2513000-s001.pdf]

Supplement 1: IRB protocol

PROTOCOL TITLE: Coordinated medical treatment of opioid use disorder and infectious disease

Coordinating opioid use treatment through medical management with infection treatment (Project COMMIT)

PRINCIPAL INVESTIGATOR: Sandra Springer, MD

Project COMMIT Study Schedule

Study Activities	Screening Process		Study Entry	Study Participation With Assigned Treatment					
	Pre-Screening	Screening	Baseline Randomization/ Induction	Wk 1	D/C	Wk 4	Wk 8	Wk 12	Wk 24
**Intervention (LAB)[d]**			X			X	X		
**Blood Buprenorphine Collection[a]**			X[b] X[b]			X[c]			
**Visit Number**		Visit 0	Visit 1	Vis2		Vis3	Vis4	Vis5	Vis 6
**Eligibility**									
Contact Primary Team	X								
Chart Review	X								
Review Inclusion/Exclusion	X								
Informed Consent		X							
Screener		X							
**Laboratory Tests/Diagnostic Procedures**									
Urine Pregnancy Test		X				X	X	X	X
Rapid HCV (confirmatory VL if applicable)			X					X	
Rapid HIV (confirmatory test if applicable)			X					X	
Urine Toxicology			X			X	X	X	X
BMP (SOC)			X					X	
CBC (SOC)			X					X	
INR (SOC)			X						
LFTs (SOC)			X					X	
HBV Core Ab, Hep B surface Ab, Surface Ag and reflex HBV DNA viral load for positive			X						
HIV VL (HIV+)			X					X	
CD4+ Count (HIV+)			X					X	
HCV VL (HCV+)			X					X	
**Interviews/Questionnaires**									
Demographics Form			X						
MINI (SUD, DSM-V d/o)			X						
AUDIT			X						
ASRS (for ADHD)			X						
WHOQOL-Bref			X			X	X	X	X

Study Activities continued	Screening Process		Study Entry	Study Participation With Assigned Treatment					
	Pre-Screening	Screening	Baseline Randomization/ Induction	Wk 1	D/C	Wk 4	Wk 8	Wk 12	Wk 24
PHQ-9			X			X	X	X	X
PCL5 (PTSD)			X						X
Modified PEG Pain Scale			X			X	X	X	X
Sexual Risk and IDU Risk Behaviors			X			X	X	X	X
Criminal Justice Questionnaire			X			X	X	X	X
Interpersonal Violence			X			X	X	X	X
Timeline Followback			X			X	X	X	X
Covid-19 Questionnaire			X			X	X	X	X
<u>Clinical</u>									
Medications			X		X	X	X	X	X
ID Questionnaire			X		X	X	X	X	X
Substance Use Treatment (SUTx) Form					X	X	X	X	X
COWS (as needed including after baseline to start on LAB)			X	X		X	X	X	X
Elixhauser Comorbidity Index			X						
<u>Safety</u>									
Modified Systematic Assessment for Treatment Emergent Events (SAFTEE) with sedation/overdose information				X		X	X	X	X
Ramsay Sedation Scale			X	X		X	X	X	X
<u>Other</u>									
Implementation Qualitative Interview (study providers, nurse care managers)*								X	X

a. Only for patients randomized to LA-B.
b. There will be two separate blood buprenorphine sample collections around the first Sublocade® dose. The first collection will be 1 hour prior to initial Sublocade® dose up to 8 hours after. The second collection should occur between 24-48 hours post the initial Sublocade® dose (up to 7 days post-dose).
c. To occur 28 days (+/- 7 days) post the initial Sublocade® dose. Must be collected prior to the second Sublocade® dose.
d. Sublocade injections may be given off study schedule according to provider clinical discretion.

*Interview will be conducted at a time point between week 12 and 24 post-randomization for selected participants.

1.0 Objectives / Specific Aims

This study seeks to test a new model of care (ID/LAB) in which opioid use disorder (OUD) is managed by infectious disease (ID) specialists and hospitalists concurrent with management of the OUD-related infections, using long-acting injectable buprenorphine (LAB), followed by referral as soon as possible after hospital discharge to community resources for long term treatment of OUD.

Specific Aims:
Aim1: The primary outcome will be a binary indicator of whether a patient is enrolled in and receiving effective medication treatment for OUD (buprenorphine, methadone, or injection naltrexone) at 12 weeks (3 months) after randomization.
Aim 2: Evidence of improved opioid use outcomes (lower days of using opioids, negative urine opioids).
Aim 3: Have higher rates of completion of the antimicrobial regimen for their infectious disease, decreased re-hospitalizations and emergency room presentations related to either their infectious disease or OUD over the 12-week follow-up period, and improved measures of quality of life.

Hypothesis: Assignment to the ID/LAB arm (OUD managed directly by the ID or hospitalist team with long-acting injection buprenorphine) will promote greater enrollment in effective medication treatment for OUD at 12 weeks after randomization, compared to TAU.

2.0 Background

The opioid use disorder (OUD) epidemic is a pressing public health crisis. In 2016, more than 64,000 Americans died of drug overdose, surpassing the number of HIV deaths at the peak of the AIDS epidemic.[1] The current OUD epidemic also results in serious infections due to injection drug use (IDU), magnifying morbidity and mortality. Hepatitis C virus (HCV), HIV infections, hospital admissions due to invasive bacterial infections including Staphylococcus Aureus bacteremia, endocarditis, skin and soft tissue infections, and bone and joint infections have all increased.[2-4] This population with co-occurring IDU-related infections represents the most severely ill OUD patients, and an important opportunity to intervene, both to improve patients' outcomes, and to reduce the public health risk of infectious disease (ID) transmission.

Currently, treatment of patients with co-occurring OUD and ID is often impeded by inadequately treated OUD. Most hospitals, particularly in under-resourced and rural areas, lack physicians trained in treatment of OUD, and the care offered for these patients typically consists of detoxification and referral to outpatient resources for follow-up treatment. This asks patients with severe OUD to tolerate withdrawal symptoms, risking premature exit from hospital, and relapse to opioid use after failure to connect with OUD treatment referrals. Results include long lengths of stay due to concern about relapse and non-adherence if patients leave the hospital, readmissions after OUD relapse, and lack of antibiotic adherence and reinfection, all leading to both poor clinical outcome and high healthcare costs. Hospital settings that manage these infections are treating increasing numbers of people with untreated OUD. This provides an opportunity to engage patients in treatment of their OUD while managing their infections. Infectious Disease (ID) specialists and hospitalists are a critical and logical resource to build capacity and increase access to medications for opioid use disorder (MOUD). With successful co-treatment of addiction and infectious diseases, OUD could be stabilized, while repeat infections are avoided, and risk of morbidity and mortality due to infection or overdose reduced. Exploring models for co-treatment of infections and OUD thus seems a promising avenue for addressing this complex dimension of the opioid epidemic.

An injectable long-acting formulation of buprenorphine (LAB), which produces therapeutic blood levels for a month, has been recently FDA-approved, and has potential advantages for initiating OUD treatment within hospital settings, including: 1) immediate and sustained treatment of opioid craving and withdrawal symptoms which frequently cause patients to leave against medical advice; 2) involvement of clinicians treating the infections in providing treatment for OUD such that treatment begins early, without waiting for addiction specialist consultation or depending on referral to treatment after hospitalization; 3) providing a bridge to long term treatment for OUD,

reducing opioid relapse and improving completion of antimicrobial treatment. This would seem particularly important for individuals in rural areas where there is limited access to specialty care.

We propose to test a new model of care (ID/LAB) in which opioid use disorder (OUD) is managed by infectious disease (ID) specialists and hospitalists concurrent with management of the OUD-related infections, using long-acting injectable buprenorphine (LAB), followed by referral as soon as possible after hospital discharge to community resources for long term treatment of OUD. Adults admitted to hospital for infections related to OUD (N = 200) will be identified and randomly assigned 1:1 to ID/LAB or treatment as usual (TAU). For this study, TAU will constitute establishment of formal diagnosis for OUD and counseling on recommendation for medication treatment including recommendation to the primary ID/ Medicine team to consider consult for OUD treatment by the typical avenue in each given hospital setting, if available.

The primary outcome measure will be the proportion of patients enrolled in effective medication treatment for OUD (buprenorphine, methadone, or extended-release naltrexone) at 3 months (12 weeks) after randomization. Study sites will be three hospitals serving geographically diverse, mixed urban and rural communities across the Eastern U.S. **The specific aims are to determine whether patients who receive ID/LAB, compared to those receiving TAU will, be more likely at 3 months post-randomization to:**

Aim 1 (primary aim): Be enrolled in medication treatment for OUD at 3 months post randomization.

Aim 2: Evidence of improved opioid use outcomes (lower days of using opioids, negative urine opioids).

Aim 3: Have higher rates of completion of the antimicrobial regimen for their infectious disease, decrease re-hospitalizations and emergency room presentations related to either their infectious disease or OUD over the 12-week follow-up period, and improved measures of quality of life.

3.0 Intervention to be studied

This is a multi-site, randomized controlled trial of adult patients (N = 200) hospitalized with severe bacterial or viral infections (complications of HCV, HIV) related to opioid use disorder (OUD). They will be recruited upon diagnosis of their infection and randomized 1:1 to one of two models of care:

1) Infectious Disease management of OUD with Long-Acting injectable buprenorphine (ID/LAB), vs. 2) Treatment as Usual (TAU). ID/LAB is the new model in which OUD is managed by Infectious Disease (ID) specialists and/or Hospitalists concurrent with management of the infectious diseases, using long-acting injectable buprenorphine (LAB). TAU is designed to systematize what is the current practice at the participating hospitals and in most U.S. hospitals while offering a minimum standard of care. TAU will constitute recommendation for MOUD initiation and consultation for addiction medicine when available; in practice, it is typically detoxification from opioids and referral to community-based addiction treatment after hospital discharge. Both models have as their goal stabilization of the OUD and establishing the patient in effective community-based medication maintenance treatment (buprenorphine maintenance or methadone maintenance) for the long term. The primary outcome measure will be the proportion of patients enrolled in effective medication treatment for OUD (buprenorphine, methadone, or extended-release naltrexone) at 3 months (12 weeks) after randomization. Study sites will be three hospitals serving geographically diverse, mixed urban and rural communities across the Eastern U.S.

Randomization: The randomization scheme will be designed by the study statistician and programmed into the data management system. Participants will be randomized to one of the two study arms (ID/LAB vs TAU) using blocks of 4, stratified by Study Site. As soon as a consenting patient has been determined to be eligible, a member of the study team will enter the data system, register the participant, obtain a unique study ID number, and the randomized group assignment will be generated by the system (ID/LAB or TAU). Treatment under the assigned study arm will then begin.

The FDA approved an injectable monthly formulation of buprenorphine (Sublocade®) on November 30, 2017. Sublocade® is indicated for the treatment of moderate-to-severe opioid use disorder (OUD) and is administered in the subcutaneous tissue of the abdomen. The manufacturer is Indivior, Inc.

Long-acting formulations of buprenorphine represent a potential breakthrough in the treatment of OUDs, reducing the risk of treatment drop-out and clinical relapse. Long-acting formulations of medications in general have been associated with better medication adherence when compared to their oral counterparts[5-8]. Probuphine®, implantable buprenorphine rods (80 mg per rod, 4 rods are implanted) deliver 6 months of treatment, but at modest blood levels and its FDA approval is for maintenance treatment of patients who are stable on 8mg/day or less of sublingual buprenorphine, not for acute treatment of OUD.[9] The FDA recently approved an injectable monthly formulation of buprenorphine (Sublocade®) that is administered in the subcutaneous tissue of the abdomen. Two doses are available, 100mg and 300mg. The 300mg dose delivers a far more adequate steady state blood level of 6.54 ng/mL over 28 days, and has been associated with better opioid abstinence outcomes for those who inject heroin[10]. Available short-term data support the hypothesis that the injectable would be superior to SL formulation. Data presented to the FDA by Indivior, the manufacturer of Sublocade®, show it is well tolerated and associated with 64-66% retention in treatment at 12 months as compared to only 34% on placebo[10].

For this study, the 300 mg dose will be the starting dose, as it produces blood levels equivalent to the higher dose range of sublingual buprenorphine, and may be more effective among heroin users. For subsequent doses the 100 mg dose can be chosen per clinical judgement (e.g. if significant side effects), although the default will be to continue the 300 mg dose as higher doses/blood levels typically translate into greater clinical efficacy.

Risks of Sublocade® include: increased opioid withdrawal symptoms if the medication is initiated too soon after last opioid dose; opioid related side effects--e.g. nausea, constipation, sedation, respiratory depression (particularly when combined with alcohol or other sedating drugs); side effects related to subcutaneous injection, pain, inflammation, or (rarely) infection at the injection site; worsening of opioid use disorder if the medication does not prove effective or is discontinued by the patient, including functional impairment as well as risk of death from drug overdose.

Treatment As Usual (TAU): There is significant practice variability across the US with regards to inpatient OUD management. For a significant amount of institutions, particularly in rural settings, so called ‘detoxification’ from opioids with non-opioid agonist medications and referral to outpatient treatment is standard clinical practice. Hence there is a gap between common practice and current best practices, which recommend initiation of MOUD for withdrawal treatment, continued therapy, and transition to the community for long term maintenance. This study will utilize a semi-structured TAU design that ensures a minimum standard of care and also reflects both variations in clinical variability and pragmatic clinical practice. TAU will constitute establishment of formal diagnosis for OUD and counseling on recommendation for medication treatment including recommendation to the primary ID/ Medicine team to consider consult for OUD treatment by the typical avenue in each given hospital setting, if available. Both control and intervention arms will receive opioid overdose education. Note: We considered allowing TAU to remain unstructured, but decided it would be best to specify TAU as we have done, for both scientific and ethical reasons. This is analogous to *D'Onofrio et al.'s* study of initiation of sublingual buprenorphine in the emergency department[11], where the design included a “facilitated referral” control condition that offered a structured brief intervention in addition to a straight referral (usual care) condition. TAU as specified provides a minimal standard of care that is clinically credible, and a comparison condition that is relatively consistent across sites.

4.0 Study Endpoints

Primary Outcome Measure--Retention in Medication Treatment for OUD: Enrollment in effective medication treatment for OUD (either buprenorphine maintenance, methadone maintenance, or extended-release naltrexone) will be ascertained through interview of the participant at each assessment point, using a modified, brief version of the Treatment Services Review[12] that records type and dose of medication treatment, contact information on the treatment program, and psychosocial treatment modalities accessed since the previous visit (e.g. professional counseling, 12-step group participation). At the key 12-week outcome point, we will ask for a release of information to contact the treatment program to verify medication type and enrollment. The primary outcome will be a binary indicator of whether or not the patient is enrolled on buprenorphine maintenance treatment or other effective medication (methadone maintenance or extended-release naltrexone) at 12 weeks after randomization, verified by either report from the treatment program, or if the treatment program does not respond, prescription drug monitoring report or EMR. Substantial evidence from longitudinal studies suggests that being on effective medication treatment for OUD is essential to maintaining abstinence, and risk of relapse to opioid use is high if buprenorphine or other medication treatment is discontinued.[13,14]A binary indicator, while it sacrifices some information, has the virtue of having a straightforward clinical meaning, particularly in this trial where the goal is to determine whether the ID/LAB model, where the medical team directly manages OUD with LAB during hospitalization, increases the likelihood of transitioning successfully to medication maintenance treatment in the community. We will examine other measures of retention as secondary outcomes, such as total weeks engaged in medication treatment, pattern of retention in treatment across time, and participation in psychosocial treatment modalities. We considered as an alternative a primary outcome measure reflecting opioid use (e.g. opioid free weeks or abstinence as end of study often favored by the FDA, or opioid relapse as used in the in the X:BOT trial[15] and in an XR-NTX vs. TAU CJS trial.[16] However, the primary goal of the models of care being tested is to secure transition into long term-community based medication treatment for OUD. The opioid outcomes are likely to be strongly associated with continued MOUD treatment and will be key secondary outcomes.

We anticipate continuing enrollment and analyses concurrently for the first three years of the study. We will continue enrollment of new participants until month nine of year 4—to ensure that the great majority of participants have completed the six-month study and that all participants will be followed for a minimum of three months post-randomization.

5.0 Inclusion and Exclusion Criteria/ Study Population

Inclusion Criteria: 1. Adult volunteers over the age of 18 who are able to provide written informed consent in English or Spanish; 2. Current hospitalization with a suspected, or known bacterial or viral (HIV/HCV/HBV) infection including but not limited to bacteremia, Candidal fungemia, osteomyelitis, endophthalmitis, septic thrombophlebitis, infected pseudoaneurysm, community acquired pneumonia, endocarditis, skin/soft tissue infection (SSTI), or septic arthritis; 3. Current moderate-to-severe OUD (DSM-5); 4. Willing to accept assignment to either ID/LAB or TAU, and to participate in research follow-up visits. Exclusions: 1. Severe medical or psychiatric disability making participation unsafe (e.g. imminent suicide risk); 2. Pregnancy, planning conception, or breast-feeding for female participants; 3. Allergy, hypersensitivity or medical contraindication to buprenorphine; 4. Moderate-severe liver impairment in the judgment of the study investigator; 5. Preexisting enrollment on methadone or buprenorphine (SL-B) maintenance AND intending to remain on methadone or buprenorphine maintenance upon discharge (patients already under effective treatment for OUD do not represent the target population of untreated OUD patients entering hospitals, nor would we want to disrupt established effective treatment). 6. Inability or unwillingness of subject to give informed consent.

A screening form that includes chart review and assessment of the above inclusion and exclusion criteria will be used to identify eligibility for the study. This form is located in the appendix.

Eligibility Screening:

The study team at each Site will conduct regular outreach to the staffs of these services to make them aware of the study and how to refer patients, and work with the attending physician to determine likely eligibility. In many cases, potentially eligible patients will be admitted to the ID or

Hospitalist service that is conducting the study. Research assistants will conduct a daily chart review of the patient lists for the Infectious Diseases and Hospitalist teams via the electronic medical record to pre-screen for eligibility. In addition, informational study flyers will be distributed to the Infectious Disease, Addiction Medicine (if applicable) and hospitalist teams with the contact information of the project coordinator for referral of participants or can contact the study team via electronic health record (EPIC) Inbox messaging. Potential participants will be asked if they would like to meet with our study staff. They will be reassured that involvement or non-involvement in the study will not have any bearing on their treatment or hospital stay. All potential participants will be screened in a confidential setting using the computer-assisted interview program.

The Gender and Minority breakdown will vary per study site. These proportions are consistent with the clinical population of opioid users in each region. It is estimated that the sample for our principle recruiting sites will be:

- Hershey, PA: 60% Male, 91% Caucasian, 2% Black or African-American, 4.8% Asian, and 1.4% more than one race; Ethnically 1.5% Hispanic or Latino
- Greenville, SC: 70% Male, 67% Caucasian, 9% Black or African-American, 3% Asian, and 10% more than one Race; Ethnically 7% Hispanic or Latino.
- New Haven, CT: 70% Male, 73% Caucasian, 20% Black or African-American, and 7% Other or More than one Race, Ethnically 27% Hispanic or Latino.

Children will not be included in this study.

6.0 Number of Subjects

N = 200 total (N = 100 per study arm), approximately 67 per site-region.

7.0 Setting

Patients eligible for the study will typically be admitted either through the emergency department or directly to the medical wards. The study team at each Site will conduct regular outreach to the staffs of these services to make them aware of the study and how to refer patients, and work with the attending physician to determine likely eligibility. In many cases, potentially eligible patients will be admitted to the ID or Hospitalist service that is conducting the study.

Yale New Haven Hospital sites:

The 944-bed Yale-New Haven Hospital (YNHH), encompassing the York Street and St Raphael campuses in New Haven, CT, serves this medium sized city and its surrounding suburban towns as well as being a tertiary care referral center for the southern New England region. The New Haven hospitals noted above will act as the primary recruiting sites in Connecticut. Bridgeport Hospital, also a member of the YNHH health system, is a 501-bed hospital which serves the southwestern region of the state, and will be a recruiting sub-site, led by Dr. Jenna Ulrich. Dr. Ulrich is an internal medicine physician, with addiction medicine experience, sees hospitalized patients and outpatients through the Primary Care Center in Bridgeport. All of these participating hospitals are heavily affected by the opioid epidemic. The Infectious Diseases Section of the Department of Internal Medicine, based at YNHH, is comprised of 36 full-time Faculty, including MPI Dr. Springer, who will lead the research team there, and 5 others with a Waiver to prescribe buprenorphine. The ID service sees over 200 consultations every month at the York Street and St. Raphael campuses in fiscal year 2017, including 8.5/month MSSA and 14.3/month MRSA bacteremia, often IDU related. The YNHH ID service itself does not have a current standard care for persons with OUD. There is a newly established Addiction Medicine consult team that is available at YNHH. ‘Treatment as usual’ ranges from Addiction medicine consultation with possible initiation onto MOUD, to no Addiction consultation followed by detoxification from opioids while inpatient.

St. Mary’s Hospital:

Saint Mary's Hospital, owned by Trinity Health of New England is a 347-bed hospital located in Waterbury, Connecticut, in the Naugatuck Valley. This region has been impacted greatly by the opioid epidemic, including associated opioid-related hospitalizations and infections. Dr. Arjet Gega is an infectious disease specialist with expertise in HIV and HCV disease management and will be the local site investigator. He sees patients both in the hospital and in his outpatient clinic. Current standard care for the OUD for these patients consists of detoxification and referrals for outpatient treatment after hospital discharge.

Columbia and Mid Atlantic site:

The Penn State Health Milton S. Hershey Medical Center is a 548-bed level I Trauma hospital and the primary academic teaching site for the Penn State College of Medicine. The medical center serves a catchment area that includes the town of Hershey, nearby Harrisburg, and the general region of rural Southeastern Pennsylvania. As elsewhere across the rural U.S., these areas have seen a substantial increase in OUD in recent years. Dr. Meredith Schade, an ID specialist, will lead the research team there in collaboration with the CTSA/CTN research team at Columbia (Dr. Nunes and colleagues). Current standard care for the OUD for these patients consists of detoxification and referrals for outpatient treatment after hospital discharge.

MUSC and SC site:

Prisma Health includes the main safety net hospital (Greenville Memorial Hospital, GMH) and is the main healthcare resource for the 11-county upstate region of South Carolina and beyond, a largely rural area including counties such as Greenville, Pickens, Oconee, and Anderson, that have been particularly hard hit by the OUD epidemic. Dr. Litwin, a General Internist, Professor and Vice Chair in the Department of Medicine at South Carolina School of Medicine and Prisma Health, who has a career focus on co-occurring OUD and HCV, will lead the study at GMH in collaboration with the CTSA/CTN research team at MUSC (Dr. Brady and colleagues). In 2017, GMH admitted over 5,000 patients with severe infections, often OUD-related, managed by ID specialists and hospitalists. Current standard care for OUD is detoxification and consultation with a social worker who provides referral for care after discharge either to the Phoenix Center, a local addiction treatment program that offers SL-Bup maintenance, or Prisma Health Recovery Program.

8.0 Recruitment Methods

Patients eligible for the study will be admitted either through the emergency department or directly to the medical wards. The study team at each Site will conduct regular outreach to the staffs of these services to make them aware of the study and how to refer patients, and work with the attending physician to determine likely eligibility. In many cases, potentially eligible patients will be admitted to the ID or Hospitalist service that is conducting the study. Research assistants will conduct a daily chart review of the patient lists for the Infectious Diseases and Hospitalist teams via the electronic medical record to pre-screen for eligibility. In addition, informational study flyers will be distributed to the Infectious Disease, Addiction Medicine (if applicable) and hospitalist teams with the contact information of the project coordinator for referral of participants. Potentially eligible patients will be given a study information sheet and asked if they are interested in considering participation. It will be stressed that participation is voluntary and that those not interested will receive standard care for their medical problems and OUD--detoxification, opioid overdose education, and recommendations and assistance with referral to community-based addiction treatment upon hospital discharge. Patients who express interest in learning more about the study will have a consent discussion with a member of the study team or one of the physicians working on the study, and provide written informed consent. Participants will complete a brief 'Consent Quiz' confirming comprehension. A member of the study team will then complete baseline assessments; medical evaluation for study medication treatment, safe study participation and eligibility confirmation. Eligible, interested participants will then be randomized.

A study information sheet will be created before recruitment begins and shared with each potential participant and will be used to recruit subjects. The brochure will explain why they are being recruited (because they are hospitalized for an infection), the purpose of the study, how the study may benefit them, and how study randomization works. The Yale Center for Clinical Investigation (YCCI)'s Recruitment and Marketing unit will assist Yale investigators in creating a brochure that is applicable to each study site.

9.0 Consent Process

Participants will be provided with a study information sheet and will only be approached for a consent discussion if they express interest in learning more about the study. We expect most potential participants to be hospitalized, so consent will take place in a private setting in the hospital, such as their room. Patients who express interest in learning more about the study will have a consent discussion with the evaluating study physician, RA, or Nurse Coordinator. They will review the study with the potential participant, provide the consent form document for the potential participant to read, discuss the study answering any questions.

The Consent Form is long and detailed, and consists of a consent form and HIPAA authorizations. Our consent procedure is designed to ensure ample time to understand and discuss its content prior to completing the informed consent process. A Consent Summary is provided at the beginning of the consent form, and the research staff member consenting the participant will walk the patient through the summary and provide an overview of the consent form. The patient will then be given the time necessary to read the consent form and write down any questions. At least 20 minutes will be set-aside for this. The research staff member will then reconvene with the patient to review the Consent Form with the patient and address any questions or concerns.

Potential study participants who remain interested after review of the consent form will be given comprehension questions to ascertain their understanding of the study, including its purpose, procedures involved, and the voluntary nature of participation. For participants who do not correctly answer all comprehension items, research staff will re-explain the study, with a focus on aspects candidates did not understand. Participants may attempt the consent comprehension questions a maximum of three times before they are deemed unable to comprehend the study. Any participant who cannot demonstrate appropriate understanding of the study to research personnel will be ineligible to participate and will be assisted in finding other treatment resources if desired.

Study participants who demonstrate understanding of the study and who voluntarily agree to participate will be asked to sign the IRB-approved consent form that they reviewed and a copy of the signed consent will be given to the participant.

Please see Appendix A for potential alterations in consent processes, including if restrictions in research are in place due to the covid-19 pandemic.

10.0 Study Design / Methods

Design Overview: This is a multi-site, randomized controlled trial in which adult patients (N = 200) hospitalized with known, or suspected, severe bacterial or viral infections (complications of HCV, HIV) and concurrent opioid use disorder (OUD) will be recruited upon admission to hospital and randomized 1:1 to one of two models of care:
1) Infectious Disease management of OUD with Long-Acting injectable buprenorphine (ID/LAB), vs. 2) Treatment as Usual (TAU). ID/LAB is the new model in which OUD is managed by Infectious Disease (ID) specialists and/or Hospitalists concurrent with management of the infectious diseases, using long-acting injectable buprenorphine (LAB). TAU is designed to systematize what is the current practice at the participating hospitals and in most U.S. hospitals while offering a minimum standard of care. TAU will constitute recommendation for MOUD

initiation and consultation for addiction medicine when available; in practice, it is typically detoxification from opioids and referral to community-based addiction treatment after hospital discharge. Both models have as their goal stabilization of the OUD and establishing the patient in effective community-based medication maintenance treatment (buprenorphine maintenance or methadone maintenance) for the long term. The primary outcome measure will be the proportion of patients enrolled in effective medication treatment for OUD (buprenorphine, methadone, or extended-release naltrexone) at 3 months (12 weeks) after randomization. Study sites will be three hospitals serving geographically diverse, mixed urban and rural communities across the Eastern U.S.

Randomization: The randomization scheme will be designed by the study statistician and programmed into the data management system. Participants will be randomized to one of the two study arms (ID/LAB vs TAU) using randomly permuted blocks of 4 stratified by Study Site. As soon as a consenting patient has been determined to be eligible, the Study Coordinator at the Site will enter the data system, register the participant, obtain a unique study ID number, and the randomized group assignment will be generated by the system (ID/LAB or TAU). Treatment under the assigned study arm will then begin.

<u>Buprenorphine and LAB initiation</u>:
For participants randomized to LAB, opioid withdrawal symptoms will be assessed on the initial post-screening visit via the Clinical Opioid Withdrawal Scale (COWS). Once a patient is 1) exhibiting clinical signs of mild opioid withdrawal, or 2) screened in with a diagnosis of OUD but with no active dependence/withdrawal, induction with sublingual buprenorphine can begin. Sublingual buprenorphine induction and dose stabilization will take place over one to two days, as per manufacturer approved accelerated protocol. Our induction period onto LAB will be more accelerated than the 7-day duration described in the Sublocade® package insert, but in line with published induction periods for other FDA approved LAB formulations.[17] Accelerated induction is crucial to the overall pragmatic model of this study in which we aim to efficiently initiate MOUD in hospitalized patients without delaying discharge. The theoretical risks of this more rapid induction protocol are potential under dosing of buprenorphine and residual withdrawal symptoms. Another possible risk is that the dose delivered by the injection buprenorphine is too high, and patient experiences opioid side effects. However, this is unlikely given participants will typically be users of intravenous opioids, likely tolerant to high doses of opioids. This will be fully mitigated given that most participants will be inpatient for their induction and will have access to medical monitoring and additional doses of SL buprenorphine if needed. For additional guidance, please refer to the Study Operations Manual.

Sublingual buprenorphine titration will follow the induction recommendations as per SAMSHA guidelines up until a max dose of 32 mg, with flexibility for clinically appropriate titration by the research teams.[18] In addition, over sedation will be assessed as well by the hospital team with assistance from the Nurse Care Manager; however we expect few to have this symptom as the participants will be actively addicted to opioids typically upon admission and thus not likely to experience opioid agonist side effects. Once felt clinically appropriate (typically day 3), participants will receive the first LAB administration of 300 mg subcutaneously injected into the abdomen by the research team Nurse Care Manager. Concurrent with the protocol for PICC line insertion, we will require patients starting injectable buprenorphine to demonstrate clearance with negative blood cultures for at least 48 hours before buprenorphine is administered. Thus SL buprenorphine may need to be continued until blood cultures are cleared prior to first LAB injection.

Administration of study Sublocade® is not always feasible prior to hospital discharge, and as such, patients randomized to ID/LA-B who are unable to start, or complete, their inductions as inpatients will be offered the opportunity to conduct their buprenorphine inductions, and receive study Sublocade®, as an outpatient. When applicable, sites may coordinate with community prescribers for routine guideline-based and clinically indicated induction onto sublingual buprenorphine. After sublingual buprenorphine inductions have been completed, any research

study Sublocade® administrations will be performed by study team members in the designated research offices and clinical spaces. Patients who receive their initial Sublocade® dose in the outpatient setting will be assessed for opioid side effects every thirty minutes, for two hours, prior to leaving the research offices and clinical spaces, which is consistent with safety procedures for patients who receive Sublocade® while inpatient. Initial Sublocade® injections should not be given after the week 8 timepoint to allow for timely assessment of adverse events at the next study visit.

A specific ordering form will be created by each institution such that the study drug may be ordered through the electronic medical system in a manner compliant with DEA and REMS restrictions for those randomized to the LAB/ID group. It is preferred that the Infectious Disease consult or primary hospitalist team writes the orders for the SL buprenorphine and LAB medications with the close guidance of the research team, in particular those providers that have an active exemption waiver for prescription of Schedule III substances as per the DATA 2000 Act (21CFR §1301.28). All involved primary providers at all sites will receive a short training presentation by the research team regarding the fundamentals of buprenorphine, side effects, drug-drug interactions, and induction dosing. Federal law states that inpatient providers are exempt from waiver requirements for maintenance or withdrawal treatment if the patient is admitted for reasons that are not directly related to withdrawal (Administering or dispensing of narcotic drugs, 21 CFR § 1306.07). By definition all patients enrolled in this study are primarily hospitalized for infections complicating their OUD and hence limited SL prescribing by non-waivered providers would be in line with federal law. If the primary ID or hospitalist team defers the specific role of buprenorphine order entry, each site's research team will have one waivered provider to be able to fulfill this function.

One of the most successful models for implementing buprenorphine in medical practices has been the Nurse Care Manager Model,[19,20] in which nurses, collaborating with physicians, share the load of evaluating and following patients with OUD. This protects the physicians' time and gives the patients the attention that they need. In the ID/LAB model the Nurse Care Manager will follow patients clinically from screening and evaluation onward (twice weekly at a minimum during inpatient, weekly outpatient until referral is secured) using the <u>Medical Management</u> counseling model. MM has been widely employed in opioid and alcohol medication trials.[21,22,23,24] In the ID/LAB model, MM will focus on <u>adherence</u> to medication treatment (both for the infections and the LAB for OUD), medication effects (including adverse events), <u>abstinence</u> from illicit opioids and other drugs and alcohol (accepting minimal use if abstinence is not the patient's goal), and <u>engagement</u> in community-based counseling and treatment resources. The Nurse Care Manager will work with the patients to transition their OUD care from the ID/LAB team during hospitalization to a community-based treatment program for ongoing medication treatment (buprenorphine, methadone, or injection naltrexone). Nurse care manager and visits will be funded by the grant, and study medications provided free of charge. With respect to sustainability, studies of implementation of the Nurse Care Manager model, suggest that third party billing can cover the cost of a nurse care manager once the service is up and running and we would expect the LAB would be covered by third parties if found to be effective in this context.

2) Treatment As Usual (TAU): The TAU arm is designed to standardize current usual care at the participating hospitals; however, services for treatment of OUD vary based on regional and institutional capability. The emerging standard of care is medical withdrawal treatment with opioid agonist therapy and transition to maintenance therapy with one of the medications for OUD (e.g., methadone, SL buprenorphine, or extended-release naltrexone) and referral to linkage on discharge. Addiction medicine consultation is another high value resource for OUD management, but availability is typically limited to tertiary care centers.

For this study, TAU will constitute establishment of formal diagnosis for OUD and counseling on recommendation for medication treatment including recommendation to the primary ID/ Medicine team to consider consult for OUD treatment by the typical avenue in each given hospital setting, if available. In addition the TAU group along with the ID/LAB intervention group will both be offered

opioid overdose education. The Nurse Care Manager will work with the primary team and participants on a schedule similar to the ID/LAB group, providing additional counseling on abstinence and discussing the importance of engagement in medication treatment for OUD which may include MOUD treatment based on the provider's choice and institution's policies. Referral to OUD treatment linkage on discharge will be available to all participants regardless of intervention designation.

For institutions with an addiction medicine consult service, the Nurse Care Manager will make the primary teams aware of their availability. Decision for consultation will be up to the primary providers based on the details of the hospitalization (i.e. plans for discharge) and the patient's amenability. If consultation takes place, decisions on withdrawal treatment, MOUD initiation and transition to community services will be deferred to the consulting team. The Nurse Care Manager will still continue to work with the primary team and participants on a schedule similar to the ID/LAB group for counseling on abstinence, overdose education, and engagement in community-based counseling.

Please see Appendix A for potential alterations in study methods if restrictions in research are in place due to the covid-19 pandemic.

Study Assessments/Procedures

A variety of laboratory analyses and questionnaire-based study instruments will be used in this study, summarized below. Appendix A contains potential alterations in laboratory or study assessments if restrictions in research are in place due to the covid-19 pandemic.

Laboratory: Rapid oral HIV1/2 Antibody test[25] or HIV P24ag/antibody serum test with confirmatory viral load for those who test positive; Rapid HCV Antibody test or serum HCV antibody with confirmatory serum viral load if positive; serum HIV viral load and CD4 for known HIV positive participants; serum HCV viral load for known HCV positive participants; Hepatitis serum surface antigen (HbSAg), surface antibody (HBSAb), core antibody (HbCAb), and quantitative DNA PCR if surface antigen is positive; point-of-care urine pregnancy test for female participants of childbearing age; point-of-care urine toxicology test.

Laboratory Specifics:

A rapid test for HIV[25] or HIV P24ag/antibody serum test will be performed on all consented participants for whom HIV status is not known at time of baseline interview. For the HIV test, participants will receive information on the procedure, meaning of test results and explanation of the window period during which an HIV antibody test might be negative (providing this information takes less than 5 minutes[26]). A reactive rapid test will be followed by a confirmatory blood test. HIV antibody testing positivity will be followed with a reflex HIV viral load We have operated routine HIV testing in previous studies for decades and have extensive experience not only complying with state laws for HIV testing, but with post-test counseling and ensuring engagement in HIV care, as indicated. In addition, rapid HCV testing or serum HCV Antibody with reflex HCV Viral load testing will also be carried out for those with unknown HCV status. Positive rapid results will be confirmed though confirmatory blood tests (HCV AB, HCV VL) if used. Those with positive results will be linked to care. There will be at least one practicing Infectious Disease physician at each site to provide any requisite oversight for testing and referrals.

For people of child-bearing potential, a urine pregnancy test will be done at baseline prior to urine drug testing. If the test is positive, a confirmatory pregnancy test will be ordered.

Participants who are pregnant at baseline will be ineligible to participate in the study. People of child-bearing potential, who are currently pregnant or found to be pregnant upon baseline testing, should be told that they are not eligible for the study without providing a reason. The person should be referred to OB/GYN or Maternal Fetal Medicine program for prenatal care. If they contact the study team at a future timepoint and indicate that they are no longer pregnant and are interested in enrolling in the trial then the Pre-Screener may be readministered.

Urine pregnancy tests will also be done at each follow-up visit. If a participant is found to be pregnant at a follow-up visit: if taking methadone, they will be encouraged to remain on methadone treatment; those who are taking buprenorphine/naloxone formulations will be encouraged to change to sublingual buprenorphine products that do not contain naloxone. Participants who become pregnant while taking study Sublocade® will be discontinued from further administration while they are pregnant, referred for obstetrical and addiction care, and the pregnancy will be followed until an outcome is known.

Study staff will reaffirm to participants that they can remain enrolled in the study no matter the outcome of the pregnancy indicating that their MOUD may need to change or be stopped during pregnancy. Study staff will also instruct participants that they may restart the assigned study medication when they are no longer pregnant if the participant is still within the treatment period of the study. If the participant is beyond the 3-month study intervention window and within the 3-month follow period (i.e., between weeks 12 and 24), the study staff will link the participant to services in the community to re-initiate treatment.

If a participant has severe hepatic disease (i.e. severe transaminitis or advanced decompensated cirrhosis, applied per clinician discretion), they will not be included in the study. LFTs will be monitored on participants for whom they have been ordered by the primary or ID consulting team. If anyone found to have a LFT >5x the upper limit of normal, their study doctor will be notified immediately. Since this will also count as an adverse event, we will follow our DSMP protocol.

Assessments:

Demographics, Drug Use and Treatment History: This brief assessment records basic demographics such as race/ethnicity, gender, housing status (homeless vs domiciled; whether living with other drug users), insurance, employment, income, marital status, and children. It will also include drug use and treatment history such as age at onset and current use of opioids and other drugs/alcohol, history of overdose, history of substance related hospitalization, and past episodes of treatment for SUD.

Locator Form: The locator form will collect patient related information not covered in the Demographics form such as social security number and contact information of family and/or close friends. Contact information will be used if primary methods of communication such as primary phone number are unsuccessful.

Opioid and Other Substance Use

- Withdrawal symptoms: The Clinical Opiate Withdrawal Scale (COWS) is an 11-item scale used in both inpatient and outpatient settings to reproducibly rate common signs and symptoms of opiate withdrawal and monitor these symptoms over time.
- Timeline Follow-Back (TLFB)[27,28](15 mins) assesses self-reported alcohol and other drug use including opioid use route of use and form of drug, for the 30 days before baseline, and for each day over the follow up period, using calendars and

memory aids to enhance recall. The TLFB has good psychometric properties, including test-retest reliability with multiple populations, and content, criterion, and construct validity across multiple related measures.[118]

- Mini-International Neuropsychiatric Interview[29] (MINI) DSM V version 7.0.2- for OUD diagnosis and other SUD diagnoses and DSM V psychiatric disorders.
- Opioid Craving: Two additional questions will assess intensity and number of days of opioid craving using a 10-point Likert scale.
- Alcohol Use Disorder Identification Test[30] (AUDIT; 10 mins) assesses for presence and severity of alcohol use disorder

Mental Health

- MINI DSM V version 7.0.2 (see above)
- Patient Health Questionnaire (PHQ-9)[31,32](5 mins) was developed for use in primary health settings and screens for DSM psychiatric diagnosis of major depression and generalized anxiety at baseline.
- PTSD Check List-5 (PCL5) for PTSD[33] (2 mins), is a 5 question assessment that evaluates for presence and severity of PTSD symptoms.
- Adult ADHD Self Report Scale (ASRS) for ADHD symptoms [34] (4 mins) is an 18 question assessment for presence and severity of ADHD symptoms by self-report.

Quality of Life: WHOQOL-Bref[35] (5 mins) is a well validated and widely used scale for persons with substance use disorders that measures the quality of social and occupational functioning as well as other domains.

Medical/Infectious Disease Questionnaire: This form documents the completion of antimicrobial therapy and re-hospitalization for infection. The initial evaluation documents the relevant infection and medical details such as infection site, organism, and stage. It also extracts the type of anti-infective, route of administration, dose, planned duration and, at select sites, knowledge of, experience with, and attitudes related to HIV Pre-Exposure Prophylaxis (PrEP). Information collected on follow-up will be alteration of treatment plan, infection related adverse events (e.g. PICC complications, drug reaction/toxicity to anti-infective agent prescribed by non-study clinicians, etc), intervening hospitalizations, and questions related to PrEP. Current study participants, at select sites, who are beyond the hospital-discharge timepoint will be asked these PrEP questions retrospectively, at one timepoint, at the next scheduled study follow-up visit.

Severity Scoring: The Elixhauser Comorbidity Index[36] is a validated scoring index for predicting mortality based on patient comorbidity. This will be performed via chart review.

Substance Use Treatment (SUTx) Form: This questionnaire documents the details of MOUD and substance use treatment history in the intervention and TAU arms. For those participants receiving LAB, it documents date of administration, dose, location and prescribing provider. The TAU section of the questionnaire queries the variety of different forms a substance use treatment that participants may or may not receive in the community. This includes MOUD (buprenorphine, extended-release naltrexone, or methadone) prescribed by non-study related physicians, behavioral therapy (i.e. 12 step programs), and whether participants have enrolled in a community opioid treatment program (OTP).

Pain: The Modified PEG is adapted from the PEG Pain Scale[37] (5 mins), which is used to measure pain over time. Two questions have been added to the initial assessment to measure pain at time of interview and withdrawal related pain. Since many of the

infections expected in the study sample are painful, and pain is a driver of opioid use, the PEG will be explored as a secondary outcome measure, moderator and mediator.

HIV Risk: Dr. Springer's HIV Risk Behavior tool created for NIDA STTR will be used to assess sexual risk behaviors and sharing of IDU related equipment.

Criminal Justice Questionnaire (1 min)- this is a set of 3 questions that asks about probation/parole status, etc.

Interpersonal Violence this is a set of 5 questions that asks about current and past relationships to assess history and current experiences with interpersonal violence. This is derived from the existing Partner Violence Screen (PVS)[38] and the Women Abuse Screening Tool (WAST).[38]

Covid-19 Questionnaire This 9-question assessment seeks to assess: 1) testing for covid-19, 2) diagnosis of covid-19, 3) Changes in substance use and 4) Changes in infection self-management by participants due to the covid-19 pandemic.

Safety:

- Ramsay Sedation Scale [39] is a clinical assessment that grades a participant's level of sedation from 1 to 6 and is useful in assessing for excessive opioid agonist symptoms with a score of 3 or greater indicating over sedation and information temporary cessation of further opioid agonist treatment.
- Serious Adverse Events: Death (and cause of death), fatal and non-fatal overdoses, and other serious adverse events via the SAFTEE[40] as commonly defined under the FDA regulations will be recorded in binary format, and summaries of each event recorded in free text. The SAFTEE has been modified to contain the particular expected AEs for LAB, including serious opioid related adverse events such as non-fatal and fatal overdose.

Implementation Factors: Qualitative Interviews (30 mins) will be conducted by the RA/Tracker with the first 10 patients at each Site assigned to the ID/LAB model at a timepoint during the 12 to 24 week period post-randomization to elicit personal accounts of their experience with the service, its acceptability, and suggestions for improvement. Participants who are unable to complete the interview by the final study timepoint, will be offered the opportunity to complete the interview at a later and more convenient date, if they wish to do so. Providers (the physicians and Nurse Care Managers delivering ID/LAB at each Site) will be interviewed at about 6 months to a year after starting enrollment about feasibility, acceptability, barriers, and facilitators within their practices and suggestions for improvement or facilitation of implementation. An interview guide will be developed during study start-up in consultation with clinical teams, and incorporating standard implementation outcomes (Glasgow et al., 1999). The interviews will be recorded and transcribed. Using Atlas.ti© software and a coding framework developed from the interview guide, the study collaborators at the Columbia Site (Jennifer Lima and Manesh Gopaldas, MD) will systematically code transcripts of the interviews, and these will be discussed at the weekly-convened study team conference call with the MPIs, Site PIs, and local Site Study Coordinators for consensus discussion. These descriptive implementation data will be used to fine-tune the implementation of the ID/LAB model at the Study Sites, and to inform development of a brief Guideline to support dissemination of the model beyond the study.

Study Timeline

Please refer to the Study Visit Schedule, located after the Table of Contents, for a detailed schematic of all measurables and their timing

Pre Screening

As discussed above (Section 5.0, Inclusion/Exclusion Criteria/Study Populations), the research team will receive referrals for pre-screening through a variety of mechanisms including a daily chart review of the Infectious Disease/Hospitalist patient lists, weekly correspondence with Infectious Disease fellows, and electronic health record inbox messages from ID/hospitalist providers. For those that are pre-screened via chart review, we will obtain a HIPAA waiver for the ability to effectively review information regarding study relevance: documentation of opioid use and an opioid related infection.

Screening

The research team member will introduce themselves and their role and ask if the patient would be interested to discuss involvement in this clinical trial. The study information sheet will be provided and protocol for screening consent will be followed as detailed extensively elsewhere (Section 9.0, Consent Process). The Screener form will review inclusion and exclusion criteria to ensure eligibility for the study. This includes 1. Proper age verification, 2. Language verification, 3. Documentation of an OUD related infection, 4. Positive screen on the The Rapid Opioid Dependence Screen (RODS), a validated screening assessment for OUD,[41] 5. No active suicidality or other severe medical or psychiatric condition (to be adjudicated by study clinician if need be), 6. Ensuring that female patients of childbearing age are not pregnant, breastfeeding or planning pregnancy, 7. Documented negative urine pregnancy test in women of childbearing age, 8. No known allergy or hypersensitivity to buprenorphine, 9. Exclusion of those with severe liver disease (to be adjudicated by study clinician if need be), and 10. The patient is not already on MOUD with intent to continue. Once the baseline screening assessments have been performed, final eligibility can be confirmed.

Baseline/Randomization/Induction

Once initial screening and inclusion has been finalized, the research associate and Nurse Care Manager or other research clinician will begin the baseline evaluation. This will include the following (defined above):

- Study Assessments: MINI, ASRS, WHOQOL-Bref, PHQ-9, PCL5, Modified PEG Pain scale, Sexual and IDU risk behaviors, criminal justice questionnaire, Interpersonal Violence questionnaire, TLFB, Elixhauser, medical/ID questionnaire, AUDIT, covid-19 Questionnaire, and SUTx assessment.
- Clinical: COWS, Opioid Craving, Ramsay sedation scale
- Other: Demographics, Locator form
- Rapid or serum HCV AB with positive HCV viral load reflex confirmation, rapid or serum HIV p24AG/AB with positive HIV viral load reflex confirmation, CD4 and viral load for known patients with HIV, HBsAg, HBsAb, HBcAb, urine toxicology. The following labs will be collected if ordered by the clinical non-research team: basic metabolic panel (BMP), complete blood count (CBC), liver function tests (LFTs), and coagulation studies (INR).

If any of the above study assessments reveal presence of an exclusion criteria that was not initially revealed on screening, the participant will be informed and removed from the study.

Follow-Up

The following measurables will occur on each of the specified research study visits.

Week 1:

- Study Assessments: none
- Clinical: COWS, Opioid Craving, Ramsay sedation scale

Week 4:
- Study Assessments: WHOQOL-Bref, PHQ-9, Modified PEG Pain scale, Sexual and IDU risk behaviors, criminal justice questionnaire, Interpersonal Violence questionnaire, TLFB, medical/ID questionnaire, AUDIT, covid-19 Questionnaire, SUTx Form, modified SAFTEE
- Clinical: COWS, Opioid Craving, Ramsay sedation scale
- Laboratory: Urine pregnancy test, urine toxicology. The following labs will be collected if ordered by the clinical non-research team: basic metabolic panel (BMP), complete blood count (CBC), liver function tests (LFTs), and coagulation studies (INR).

Week 8:
- Study Assessments: WHOQOL-Bref, PHQ-9, modified PEG Pain scale, Sexual and IDU risk behaviors, criminal justice questionnaire, Interpersonal Violence questionnaire, TLFB, medical/ID questionnaire, AUDIT, covid-19 Questionnaire, SUTx Form, modified SAFTEE
- Clinical: COWS, Opioid Craving, Ramsay sedation scale
- Laboratory: Urine pregnancy test, urine toxicology. The following labs will be collected if ordered by the clinical non-research team: basic metabolic panel (BMP), complete blood count (CBC), liver function tests (LFTs), and coagulation studies (INR).

Week 12:
- Study Assessments: WHOQOL-Bref, PHQ-9, PCL5, modified PEG Pain scale, Sexual and IDU risk behaviors, criminal justice questionnaire, Interpersonal Violence questionnaire, TLFB, medical/ID questionnaire, AUDIT, covid-19 Questionnaire, SUTx form, modified SAFTEE
- Clinical: COWS, Opioid Craving, Ramsay sedation scale
- Rapid or serum HCV AB with positive HCV viral load reflex confirmation, rapid or serum HIV p24AG/AB with positive HIV viral load reflex confirmation, CD4 and viral load for known patients with HIV, HBsAg, HBsAb, HBcAb, urine toxicology. The following labs will be collected if ordered by the clinical non-research team: basic metabolic panel (BMP), complete blood count (CBC), liver function tests (LFTs), and coagulation studies (INR).

Week 24:
- Study Assessments: WHOQOL-Bref, PHQ-9, modified PEG Pain scale, Sexual and IDU risk behaviors, criminal justice questionnaire, Interpersonal Violence questionnaire, TLFB, medical/ID questionnaire, AUDIT, covid-19 Questionnaire, SUTx Form, modified SAFTEE
- Clinical: COWS, Opioid Craving, Ramsay sedation scale
- Laboratory: Urine pregnancy test, urine toxicology. The following labs will be collected if ordered by the clinical non-research team: basic metabolic panel (BMP), complete blood count (CBC), liver function tests (LFTs), and coagulation studies (INR).

<u>Research Data Management System</u>: A web-based, direct data entry and data management system will be developed under the direction of Richard Buchsbaum at the Columbia Department of Biostatistics. All CRFs and scheduled assessments will be programmed into the system. Research Assistant/Trackers will collect and enter the data directly in real time while interviewing patients for the Baseline and Follow-up assessments.

All databases developed and maintained at the Columbia Department of Biostatistics are stored in natively encrypted files on secure servers. The servers are secured by password-only access, software and hardware firewalls, and continuous intrusion monitoring. Communication with the servers occurs only over encrypted channels, and access to the web interface is strictly password protected (passwords must also conform to strength rules and expire every 120 days). Role-based security is also employed, meaning only users with a need to access confidential data can do so and there is institute access monitoring, so that every attempt to access confidential data is logged. The system is compliant with all relevant regulations (HIPAA, HITECH, CFR21 part 11, etc.), and is certified by the IT security team at Columbia University Medical Center. As such, the system is subject to regular audits of settings and procedures as well as security scans. The Columbia Department of Biostatistics has hosted dozens of multi-center studies, and currently hosts more than 50 projects, most which contain confidential data. There have been no data breaches within this web-based system.

Assessment Team and Procedures: Each Site will employ a Research Assistant/Tracker who will be supervised and backed up by the Site Study Coordinator who will also act as a resource. The assessment packet is designed so that it can be collected either in person at the hospital or in the field (RA/Trackers will travel to meet patients as needed) or by telephone if necessary.

Study interviews will be conducted using the web-based data entry system.

11.0 Specimen Collection and Banking (if applicable)

Optional buprenorphine blood analyses will be conducted on patients who are randomized to the LA-B study arm. Enrollment in this sub-study will be ongoing until 24 complete sample sets are collected (i.e., one blood sample collection at three timepoints for each of the 24 patients). Recruitment in the sub-study will continue until the 24th sample set is completed. At that time the 24th sample set is completed enrollment in the sub-study will stop. Participants who are mid-participation in the sub-study at the time the 24th set is completed will be allowed to finish sub-study participation, potentially leading to more than 24 total complete sample sets collected for the sub-study. If it known that a participant will be unable to complete all three sample collections, they will be withdrawn from the sub-study. Pharmacokinetic data exists for Sublocade® from Phase 3 data, however less data exists for patients in this study population (hospitalized; concurrent infection). Patients will be asked to consent to these blood draws as part of the informed-consent process. Timepoints for measurements are as follows:

Blood draw 1: 1 hour prior to initial Sublocade® dose up to 8 hours after.

Blood draw 2: 24-48 hours post the initial Sublocade® dose (up to 7 days post-dose).

Blood draw 3: 28 days (+/- 7 days) post the initial Sublocade® dose. Blood draw 3 must be collected prior to the second Sublocade® dose.

12.0 Data Management

Power Analysis: Previous studies of buprenorphine initiation in an Emergency Department[42] and on a medical inpatient service,[43] yielded rates of post-discharge buprenorphine maintenance treatment in the 70% range. The Emergency Department study implemented a Screening, Brief Intervention and Referral control condition, similar to the TAU condition planned in the current protocol, which yielded a rate of post-discharge buprenorphine maintenance treatment of 45%. We anticipate that the population recruited for this study will be among the most severely ill patients with OUD, being those requiring hospitalization for infections, and therefore we conservatively assume that the proportion of patients meeting our primary outcome (enrolled in MOUD 12 weeks after hospital discharge) will be 40% in the TAU arm and 60% in ID/LAB arm. We have chosen a sample size of N = 200 total (N = 100 per study arm), in order to ensure

sufficient power (power = 80%) with a two-sided level of significance of 5% to detect a difference of at least 19.7% between the TAU and ID/LAB treatment groups (i.e., 40% treatment enrollment in the TAU arm and at least 59.7% treatment enrollment in the ID/LAB arm). This rate difference of 19.7% is equivalent to an odds-ratio of 2.2 and would be clinically meaningful.

Data Analysis Plan:

Primary outcome: The primary outcome will be a binary indicator of whether a patient is enrolled in and receiving effective MOUD (buprenorphine, methadone, or injection naltrexone) at 12 weeks (3 months) after randomization.

Secondary outcomes: 1) Days using opioids, and days injecting drugs per 28 days prior to each major assessment point (weeks 4, 8, 12, and 24 post randomization) (longitudinal, continuous); 2) Urine toxicology-confirmed abstinent (urine toxicology negative for illicit opioids, and self-report negative over past 7 (at week 1) or 30 days (weeks 4, 8, 12, 24) (longitudinal, binary); 3) Completion of the prescribed course of antimicrobial therapy (binary), Re-hospitalization for infection by week 12 (binary); 4) Days using other drugs/alcohol per 28 days prior to each assessment (longitudinal, continuous); 5) Urine toxicology positive for non-opioid drugs (longitudinal, binary); 6) Social functioning and quality of life (WHOQOL-Bref) at 12 and 24 weeks after randomization (longitudinal, continuous); 7) Modified PEG Pain Scale at each assessment (Longitudinal, continuous) 8) HIV risk behaviors—days in past 28 days of vaginal or anal sex without a condom at weeks 4, 8, 12, 24 (longitudinal, continuous); 8) Any needle sharing in past 28 days (longitudinal, binary); 9) Treatment satisfaction (via the TSR)[12] at 12 and 24 weeks (longitudinal, continuous); 10) Discharge "against medical advice" (binary); 11) Cure of index infection (binary); 12) HCV cure or link to treatment (binary); 13) HIV Viral suppression (Viral Load < 200 copies), if HIV positive (binary); 14) overall hospitalization by week 12; 15) ED visits by week 12; 16) Serious adverse events (SAFETEE and non-fatal and fatal overdose) (binary).

Additional variables: _Covariates, primary and secondary analyses_: *Stratification factors*: Site (3 sites: YNHH; Columbia/Penn State Hershey; South Carolina/Greenville) will be included as a fixed effect in all analyses. *Covariates*: In general, the baseline score for each outcome measure (where applicable) will be included as a covariate in outcome analyses. _Moderators_: A range of baseline moderator variables will be explored including 1) Demographic characteristics (gender, age, ethnicity, living situation (domiciled vs homeless); 2) Modified Pain PEG scores at baseline (continuous) 3) Evidence of fentanyl use at admission (urine toxicology, self-report) (binary); 4) Other substance use at baseline (any cannabis, any stimulant, any heavy drinking days; binary); 5) Probable major depression at baseline (binary based on PHQ score); 5) Type of infection (endocarditis/ osteomyelitis/bacteremia/ cellulitis/ etc.). _Mediators_: 1) Social functioning (WHOQOL-Bref); continuous); 2) Opioid craving (continuous); 3) Pain (Modified PEG scores; continuous).

<u>Intent to Treat, Dropouts, and Missing Data:</u> All analyses will be on the Intent-to-treat (ITT) sample, i.e., all randomized subjects (N=200) according to the treatment arm to which they were assigned. For the primary outcome and secondary outcomes related to treatment engagement, any patients lost to follow-up will be assumed not to be enrolled in treatment nor to have completed the antimicrobial course. Loss to follow-up will be minimized through collection of extensive locator information at baseline and proactive outreach, as described above. For the secondary outcomes related to substance use, missing data (patients who cannot be located) will assumed to be opioid positive (not abstinent), a typical assumption which is reasonable based on the high rate of relapse among patients with OUD who discontinue medication treatment.[14,44-46]For other secondary outcomes, missing data will be treated as missing at random. Longitudinal mixed effects models do not require complete data to provide estimates of the outcome, and the estimates are considered valid under the assumption that the data are missing at random.[47]. We will additionally perform sensitivity analysis for the secondary outcomes to examine the influence on the outcomes of dropout and missing data by performing several imputation methods, for instance imputing missing weeks as all abstinent or all non-abstinent. Comparison of the inferences from assuming various models for the missingness provides a measure of the validity of the efficacy estimate from the initial model.

Significance Testing and Preliminary Analyses: All tests for main effects will be performed at two-sided significance level of 5%. Before performing specific analyses, we will examine the distributions of outcome measures and covariates and investigate for outliers. The distributions of continuous variables will be checked for normality, and transformed, if necessary. Distribution of demographic and other baseline variables will be examined and described in terms of means, standard deviations, proportions and 95% confidence intervals. Covariates will be examined for association with treatment outcome. Covariates associated with treatment outcome will be adjusted for in models used to test study hypotheses.

Primary outcome **Statistical Model:** *Hypothesis: Assignment to the ID/LAB arm (OUD managed directly by the ID or hospitalist team with long acting injection buprenorphine) will promote greater enrollment in effective medication treatment for OUD (MOUD) at 12 weeks after randomization, compared to TAU.* The effect of randomization to the ID/LAB arm compared to the TAU arm will be estimated using logistic regression with the binary outcome of enrollment (yes/no) modeled as a function of treatment condition (ID/LAB vs TAU), with Site as a fixed effect, and covariates/moderators as listed above under 'Additional Variables'. The odds ratio of the treatment term and its confidence limits will estimate the treatment effect. The raw retention rates in each treatment group and rate differences will be computed for descriptive purposes.

Power Analysis: Previous studies of buprenorphine initiation in an Emergency Department[42] and on a medical inpatient service,[43] yielded rates of post-discharge buprenorphine maintenance treatment in the 70% range. The Emergency Department study implemented a Screening, Brief Intervention and Referral control condition, similar to the TAU condition planned in the current protocol, which yielded a rate of post-discharge buprenorphine maintenance treatment of 45%. We anticipate that the population recruited for this study will be among the most severely ill patients with OUD, being those requiring hospitalization for infections, and therefore we conservatively assume that the proportion of patients meeting our primary outcome (enrolled in MAT for OUD 12 weeks after hospital discharge) will be 40% in the TAU arm and 60% in ID/LAB arm. We have chosen a sample size of N = 200 total (N = 100 per study arm), in order to ensure sufficient power (power = 80%) with a two-sided level of significance of 5% to detect a difference of at least 19.7% between the TAU and ID/LAB treatment groups (i.e., 40% treatment enrollment in the TAU arm and at least 59.7% treatment enrollment in the ID/LAB arm). This rate difference of 19.7%% is equivalent to an odds-ratio of 2.2 and would be clinically meaningful.

Secondary outcomes: **Statistical Models**: For each of the secondary outcomes (see above) the effect of randomization to the ID/LAB arm compared to the TAU arm will be estimated with a model of the same general form as for the primary outcome (independent variables: treatment assignment (ID/LAB vs TAU), Site as a fixed effect, and baseline score as covariate where relevant) using logistic regression models (for binary outcomes), generalized linear model (for continuous outcomes), or longitudinal generalized mixed effects model (for longitudinal outcomes) with appropriate link functions (e.g. logit for binary outcomes). Longitudinal outcomes analyzed using a mixed effects models will utilize a generalized estimating equation with an autoregressive correlation structure (AR(1)) to account for within subject correlation over time, as well a random intercept accounting for between subject variability. In addition to site, covariates that are found to be related to the outcomes will be added to the models to improve the power for detecting significant differences.

Moderation and Mediation: **Moderation Statistical Models**: The effect of randomization to the ID/LAB vs TAU arms will be estimated for subpopulations based on the demographic and baseline characteristics selected as moderators (see above). We will estimate the moderation effect on primary outcome using a similar model to Aim 1 primary outcome with the inclusion of moderator by treatment interaction: $Y_i= \beta_0+\beta_1 Trx_i+\beta_2 M_i +\beta_3 Trx_i M_i+\beta_4 C_i+s_i$ where M_i is the moderator status for i^{th} subject and Trx_i is subject's treatment assignment. Regardless of the significance of the interaction term, we will use the model to estimate the 95% confidence interval of the treatment effect for each level or category of the moderator variable, or the treatment effect at selected values of the moderator variable, if continuous. The moderator analyses are exploratory and hypothesis generating, but significant moderator effects can be useful in planning

future efforts at implementation of medication treatment for opioid use disorder in medical settings by identifying subgroups that may particularly benefit. Identifying subgroups where there is less benefit may suggest future modifications to the implementation strategy to achieve better effect in those subgroups.

Mediation Statistical Models: The effect of randomization to the ID/LAB vs TAU arms will be estimated when accounting for potential mediators (see above). In particular, we are interested in testing whether an impact of treatment on the primary outcome (enrollment in effective medication treatment at 12 weeks after randomization) is mediated by social functioning, craving for opioids, pain, or mental health symptoms. We will simultaneously estimate both the total effect of treatment assignment on primary outcome and the indirect of treatment assignment on primary outcome mediated through the intended target mediators using structural equation modeling (SEM) in software MPlus.[48]The indirect effect is estimated by the product of the effect of treatment assignment on the mediator and the effect of the mediator on the primary outcome while adjusting for treatment assignment. SEM will provide an estimate of the indirect effect of each mediator as well as its bootstrapped 95% confidence interval. These mediational analyses can be of heuristic value in suggesting aspects of the ID/LAB implementation strategy that might need strengthening in future research or implementation efforts.

Confidentiality of Data:

The data system will be unitary, hosted on secure servers at Columbia. All sites will access the same system. The system incorporates role-based security, so users only have access to data appropriate to their role, e.g. coordinators for a particular site only have access to data from their site. The system is certified by CUMC IT as secure for the storage of confidential data for research.

The screener will consist of inclusion/exclusion criteria questions only, and an ID number that is separate from their study ID number (if enrolled) will be used.

To ensure protection of confidentiality, assessment/questionnaire data will be entered into the study database with no personal identifiers and source documents will only identify participants by study ID number. Personal identifying information (including locator information) will be kept on paper in locked files at each site. Access to computers used for data entry, data management, and analysis will be password protected and limited to study investigators. All data that includes PHI will only be stored on the secure servers at Columbia mentioned above, no PHI will be stored on tablets or computers.

Access to this data will be restricted to specific study staff. This information will be collected form each participant at baseline and at follow-up visits only if contact information changes. Contact information will include the participant’s name, phone number, address, email address, aliases (if used), and alternative contacts and their contact information. Contact information, such as telephone number is important, as it will be used to remind participants of follow-up visits or to contact a participant if they are late or do not show up to a follow-up visit. Alternative contacts will only be contacted if we cannot get in contact with the participant.

All information will be stored in password-protected tablets or computers with double-password protection for opening specified files, computers have been encrypted in accordance to HIPAA guidelines. All research data not entered into the system created by Columbia will be stored in the double-locked research offices at each study site. Client level data will be stored with research study number and without any unique identifiers. All identifiers that link study number to study subject will be housed in PGP encrypted, password-protected computers available only to the Project Coordinator, Data Manager, co-Principal Investigators, and Statistician. All computers used for study purposes have been encrypted to meet all HIPAA requirements.

Effective October 1, 2017 Certificates of Confidentiality (CoCs) will be issued automatically for any NIH-funded project using identifiable, sensitive information that was on-going on/after

December 13, 2016. The CoC will be issued as a term and condition of this award. There will be no physical certificate issued.

<u>Data Monitoring and Quality Control:</u>
Monitoring for the study is provided by Columbia University, including regular data monitoring at the study sites.

Ongoing data and safety monitoring will be conducted weekly during research team meetings attended by the study site PI with the study site team, as well as weekly multi-site conference calls with all of the study sites, PIs, other research team members, and a representative from YCCI and the Data Management groups.

A Quality Assurance Monitor will ensure site readiness via a site initiation checklist and will travel to the sites to perform a complete review of the first 3-5 randomized participant records. The monitor will return to each study site biannually to review 10% of participants' study records as well the site regulatory binder. Every six months, the PIs, the study site teams, and the QA monitor will review the number of participants enrolled, the number who completed the protocol, the number who dropped out of the protocol prior to completion (and reason why), any adverse events, procedures for assuring participant privacy and confidentiality, and the quality and integrity of the data collected. Corrective action will be taken if needed. Investigators will also coordinate monitoring required by the sIRB or any other reporting entity.

No data will reside at MUSC.

13.0 Provisions to Monitor the Data to Ensure the Safety of Subjects (if applicable)

The DSMP is attached to the IRB application.

14.0 Withdrawal of Subjects (if applicable)

If the study team feels that the participant's condition is worsening, the team may terminate the participant's study treatment and institute a higher level of care, such as residential treatment or expedited initiation of medication for opioid use disorder. This is outlined in further detail in the DSMP.

As part of the informed consent process, participants will be informed that part of participation in the study means that the study team will track participants into the community who lose contact with the study team and miss appointments for research assessments, and what the tracking involves (e.g. contacting family members and other personal contacts that the participant has listed and given permission to be contacted, looking for the participant in the community in places where he/she is known to frequent). Participants will be informed that they can refuse to engage with study staff and/or ask for their study participation to end and tracking efforts will be discontinued. However, it is our experience from other treatment studies employing active tracking of this type that participants are often grateful for the effort to locate them and typically willing to engage/re-engage with the study team.

15.0 Risks to Subjects

As in all research studies, there are potential risks. We go to great efforts, however, to ensure that risks are minimized. This is particularly true as we intend to conduct this study with a vulnerable population. Moreover, there is an additional risk with this study, particularly since it initially involves research with those with substance use disorders. Within this section, we will first describe the individual risks associated with the interviews, followed by the laboratory testing, and other potential risks. There may be risks that are currently not known or have yet to be experienced by patients taking Sublocade®.

<u>Risks Associated with Opioid Use Disorder</u>: Opioid use disorder is a dangerous disorder

associated with impaired functioning, morbidity (including infections of the type that participants in this protocol will be suffering from on admission), and mortality due to opioid overdose. Patients will be protected from opioid use while inpatient, but the risk of relapse is high after discharge from hospital unless the patient is maintained on one of the effective medications (methadone, buprenorphine, or extended-release naltrexone) which protect against relapse and overdose. Participation in the research study may mitigate these risks to the extent that patients in the ID/LAB arm are started and maintained on long-acting injection buprenorphine, and patients in TAU arm started on and successfully referred to ongoing medication treatment. This risk may differ between study arms to the extent that the ID/LAB arm is more successful at stabilizing patients on maintenance medication.

Risks Associated with Questionnaires: During the administration of questionnaires, topics such as alcohol and drug use, alcohol and drug treatment, mental health and questions regarding HIV risk behaviors will come up. These topics may make participants feel uncomfortable. Participants may choose not to answer all or any part of a question. Participant names will be recorded, but kept safe, according to HIPAA regulations. If the participant chooses to be screened for the study, minimal identifiable information will be collected to ensure eligibility. Consent procedures are explained above. If the potential participant agrees to consent, they will be asked to sign an IRB approved consent form on a tablet. Data from the questionnaires will be reviewed after they have been completed. No study-related unanticipated problems or adverse events are expected to occur in this part of the study. Hard copies of data collected will be stored in a locked cabinet, although we expect minimal to no paper copies. Electronic copies can be accessed by providing a user identification and password.

Risks Associated with HIV testing: Participants will receive verbal information about rapid HIV and HCV testing including a description of the rapid testing procedure, timing for and meaning of test results and explanation of the window period during which an HIV and HCV antibody test might be negative. Providing this information takes less than 5 minutes. Participants will then be offered the OraQuick Rapid HIV-1/2 and OraQuick Rapid HCV Antibody Tests. The Research Assistants have been trained in how to perform these rapid tests. A reactive test will be followed by a confirmatory blood test. Participants who test positive will also receive emotional support and posttest counseling on sexual and injection drug use risk behavior and importance of ongoing HIV primary medical care and HCV treatments. They will be assisted in scheduling an appointment with an HIV provider. There will be at least one practicing Infectious Disease physician at each site to provide any requisite oversight for testing and referrals.

In accordance with CT, PA and SC state laws, any new HIV and HCV test results must be reported and this will be outlined in each consent form. We have operated routine HIV and HCV testing for study participants for decades and have extensive experience not only complying with state laws for HIV testing, but with post-test counseling and ensuring engagement in HIV care if indicated. All results will be kept confidential.

Risks Associated with Loss of Confidentiality: As with all research, there is a risk that involves potential breaches of confidentiality. We intend to do everything possible to reduce this risk and this is one of the process measures we propose to measure. Participants with substance and alcohol use disorders are a vulnerable population. Thus, our research to date has insured strict confidentiality safeguards.

Potential sites for breaches of confidentiality include during the recruitment process at the hospital, during study interviews, or from data management systems. From our research efforts and provision of clinical care for out-of-treatment drug users, HCV-infected and HIV infected drug users in clinical settings, we have paid careful attention to potential breaches of confidentiality for subjects undergoing interviews. All information is stored in password-protected, encrypted computers with double-password protection for opening specified files. All confidential information (study instruments, laboratory results, etc.) will be recorded with study participant number only and maintained in locked cabinets within offices at each study site; data will only be available to

be opened by the Project Coordinator, Data Manager, co-Principal Investigators, and Statistician. Electronic databases will be maintained through password-protected computers and files and maintained at the Columbia Department of Biostatistics.

Risks associated with long-acting injectable buprenorphine (LAB):
Risks of LAB (Sublocade®) include: increased opioid withdrawal symptoms if the medication is initiated too soon after last opioid dose;
Side effects related to subcutaneous injection: pain, inflammation, or (rarely) infection at the injection site. There have been some reports of severe injection-site reactions causing abscess, ulceration, and necrosis, in some cases requiring surgical depot removal, debridement, antibiotic administration;
Side effects related to opioids: nausea, constipation, sedation, respiratory depression (particularly when combined with alcohol or other sedating drugs). Opioid use disorder could worsen if the Sublocade® does not prove effective or is discontinued by the patient, including functional impairment as well as risk of death from drug overdose.

Risks associated with Treatment as Usual (TAU): The main risks associated with TAU include: opioid withdrawal symptoms during detoxification; worsening of opioid use disorder, including functional impairment as well as risk of death from drug overdose.

Risks associated with drawing blood include momentary discomfort and/or bruising. Infection, excess bleeding, clotting, or fainting is possible, although unlikely.

Risks associated with pregnancy: If a participant becomes pregnant and tests positive for illegal drugs, it is a law that the South Carolina Department of Social Services (DSS) must be notified. Participants could be ordered to mandatory drug treatment, lose custody of their children, or be jailed. We will mitigate these risks by performing all pregnancy tests before tests for illegal substances, and by making clear in the consent form these reporting requirements. A person who is found to be pregnant will be withdrawn from the study and no further urine toxicology testing or questions regarding drug use will be queried; they will subsequently be referred to care for both pregnancy and addition. There are no such reporting requirements in Pennsylvania and Connecticut.

Psychiatric Risks:
If someone has a potential new psychiatric diagnosis via any of the structured questionnaires, a study doctor will be notified within 48 hours of the assessment. The participant will be linked for an evaluation and care with a clinician from a designated Psychiatric Team at each site. Participants with existing psychiatric diagnoses will continue their treatments as usual.

If someone is actively suicidal (which can be determined by participant self-report, the PHQ suicidality question OR the MINI suicidality section), each site will follow the general protocol below:

1. If the research staff member is uncomfortable and there is a more experienced clinician available, they will request their assistance. If assistance is not available or the client reports imminent intent to harm self or others, the study staff member will:
   If the participant is currently hospitalized, a referral will be made to inpatient psychiatry.
   If participant is not hospitalized, we will use the following protocol:
   a. Have a co-worker call 911
   b. Stay with participant, not leave participant alone
   c. Escort participant to ambulance and provide all necessary information
   d. Notify study doctor, who will plan a psychiatric team consultation for participant
2. If a participant is not in an imminent danger to him/her self or others:
   a. If the participant is currently hospitalized, a referral will be made to inpatient psychiatry.
   b. If participant is not hospitalized, give client telephone numbers for crisis counseling, emergency room, or local mental health association

c. A study staff member will follow up with the participant within 24 hours
d. Notify study doctor, who will plan a psychiatric team consultation for participant

16.0 Potential Benefits to Subjects or Others

Potential benefits to participants may include improved outcome of their opioid use disorder (OUD) and infections and other medical problems, as a result of treatment of OUD provided by the study. The ID/LAB group involves treatment with long acting buprenorphine, FDA approved as effective for treatment of OUD, and the TAU group will receive a minimum of OUD diagnosis and recommendation for inpatient MOUD initiation from a Nurse Care Manager which is in many cases an enhancement over standard care. Both groups are assisted with securing effective medication assisted treatment for OUD in the community after hospital discharge.

17.0 Sharing of Results with Subjects

Results of rapid HIV and HCV testing will be shared with participants, as described above. Results will also be shared according to each state's reporting laws, which will be outlined in each site's consent form.

18.0 Drugs or Devices (if applicable)

Yale and CT Site:
We will use the Yale Investigational Drug Service (IDS) to store and supply injectable buprenorphine (Sublocade®) for each participant's injection. They will follow the standard processes for dispensing controlled substances. Our study nurse will pick up any medications from the IDS and sign the prescription pick-up log. All prescriptions will have an administration worksheet, which will be completed by the nurse upon administration. The administration worksheet will also allow for documentation of drug disposal or return, which can be faxed back to the pharmacy.

Columbia and PA Site: The GHS Research Pharmacy will be responsible for ensuring clinical trial compliance with regards to medication storage, dispensing, record-keeping, and providing clinical expertise. The Research Pharmacy provides patients, staff, investigators, and sponsors with a well-organized and properly run study that will meet all state and federal rules and regulations.
Provided services include:
• Assistance in the execution of the scientific, clinical, and administrative functions of drug research.
• Assure compliance with all federal, state, sponsor, and IRB regulations concerning medication used on investigational studies.
• Reviewing the protocol and all printed material pertinent to pharmacy dispensing
• Preparing an Investigational Drug Data sheet (1 or 2 pages of primary information about the study) that is then provided to pharmacy and ancillary staff
• Setting up the drug study in one or more settings or locations. (I.e. Outpatient, Inpatient, Physician office practices)
• Preparing the proper paperwork and records as required by the pharmacy sections of the protocol.
• Proper handling (receiving, storing, preparing and dispensing) of all study drug materials.
• Storage of all pharmacy related study records, during and after the study.
• Preparation and maintenance of blinding information.
• Preparation and maintenance of drug dispensing information (directions for proper preparation of study drug).
• Assisting with monitoring visits from study sponsors.
• Monitored and alarmed storage facilities for all drug requiring refrigeration or freezer storage.
• Invoicing for research pharmacy fees
• Proper handling of study drug when the study has finished: either returned to the study sponsor or destroyed on site following company policy and proper destruction procedures.

MUSC and SC Site: Injectable buprenorphine (Sublocade) will be shipped and stored at each Investigational Drug Service (IDS) as designated on the form FDA 1572. The IDS will follow institutional policies for storing and dispensing controlled substances.
Our study nurse will pick up any medications from the IDS and sign the prescription pick-up log or the IDS staff will deliver any medications to the study nurse who will sign the prescription pick-up log. All prescriptions will have an administration worksheet, which will be completed by the nurse upon administration. The administration worksheet will also allow for documentation of drug disposal or return, which can be faxed back to the pharmacy.

References

1. Rudd RA, Seth P, David F, Scholl L. Increases in Drug and Opioid-Involved Overdose Deaths - United States, 2010-2015. *MMWR. Morb. Mortal. Wkly. Rep.* 2016;65(5051):1445-1452.
2. Ronan MV, Herzig SJ. Hospitalizations Related To Opioid Abuse/Dependence And Associated Serious Infections Increased Sharply, 2002-12. *Health affairs (Project Hope).* 2016;35(5):832-837.
3. Conrad C, Bradley HM, Broz D, et al. Community Outbreak of HIV Infection Linked to Injection Drug Use of Oxymorphone--Indiana, 2015. *MMWR. Morbidity and mortality weekly report.* 2015;64(16):443-444.
4. Zibbell JE, Asher AK, Patel RC, et al. Increases in Acute Hepatitis C Virus Infection Related to a Growing Opioid Epidemic and Associated Injection Drug Use, United States, 2004 to 2014. *Am J Public Health.* 2018;108(2):175-181.
5. Brooks AC, Comer SD, Sullivan MA, et al. Long-acting injectable versus oral naltrexone maintenance therapy with psychosocial intervention for heroin dependence: a quasi-experiment. *J Clin Psychiatry.* 2010;71(10):1371-1378.
6. Hulse GK, Morris N, Arnold-Reed D, Tait RJ. Improving clinical outcomes in treating heroin dependence: randomized, controlled trial of oral or implant naltrexone. *Arch Gen Psychiatry.* 2009;66(10):1108-1115.
7. Leucht C, Heres S, Kane JM, Kissling W, Davis JM, Leucht S. Oral versus depot antipsychotic drugs for schizophrenia--a critical systematic review and meta-analysis of randomised long-term trials. *Schizophr Res.* 2011;127(1-3):83-92.
8. Winner B, Peipert JF, Zhao Q, et al. Effectiveness of long-acting reversible contraception. *The New England journal of medicine.* 2012;366(21):1998-2007.
9. Rosenthal RN, Lofwall MR, Kim S, et al. Effect of Buprenorphine Implants on Illicit Opioid Use Among Abstinent Adults With Opioid Dependence Treated With Sublingual Buprenorphine: A Randomized Clinical Trial. *JAMA.* 2016;316(3):282-290.
10. Slough I. INDIVIOR PLC Results from the Phase 3 Pivotal Study of RBP-6000 Buprenorphine Monthly Depot for the Treatment of Opioid Use Disorder. . Conference on Problem of Drug Dependence (CPDD); 2017; Montreal, Canada.
11. D'Onofrio G, Chawarski MC, O'Connor PG, et al. Emergency Department-Initiated Buprenorphine for Opioid Dependence with Continuation in Primary Care: Outcomes During and After Intervention. *Journal of general internal medicine.* 2017;32(6):660-666.
12. McLellan AT, Alterman AI, Cacciola J, Metzger D, O'Brien CP. A new measure of substance abuse treatment. Initial studies of the treatment services review. *J Nerv Ment Dis.* 1992;180(2):101-110.
13. Weiss L, Netherland J, Egan JE, et al. Integration of buprenorphine/naloxone treatment into HIV clinical care: lessons from the BHIVES collaborative. *Journal of acquired immune deficiency syndromes.* 2011;56 Suppl 1:S68-75.
14. Hser YI, Evans E, Huang D, et al. Long-term outcomes after randomization to buprenorphine/naloxone versus methadone in a multi-site trial. *Addiction.* 2016;111(4):695-705.
15. Lee J, Nunes E, Novo P, et al. Comparative effectiveness of extended-release naltrexone versus buprenorphine-naloxone for opioid relapse prevention (X:BOT): a multicentre, open-label, randomised controlled trial. *The Lancet.* 2017.

16. Lee JD, Friedmann PD, Kinlock TW, et al. Extended-Release Naltrexone to Prevent Opioid Relapse in Criminal Justice Offenders. *The New England journal of medicine.* 2016;374(13):1232-1242.
17. Lofwall MR, Walsh SL, Nunes EV, et al. Weekly and Monthly Subcutaneous Buprenorphine Depot Formulations vs Daily Sublingual Buprenorphine With Naloxone for Treatment of Opioid Use Disorder: A Randomized Clinical Trial. *JAMA Intern Med.* 2018;178(6):764-773.
18. TIP 63: Medications for Opioid Use Disorder For Healthcare and Addiction Professionals, Policymakers, Patients, and Families. In: SAMHSA, ed2019.
19. LaBelle CT, Han SC, Bergeron A, Samet JH. Office-Based Opioid Treatment with Buprenorphine (OBOT-B): Statewide Implementation of the Massachusetts Collaborative Care Model in Community Health Centers. *J Subst Abuse Treat.* 2016;60:6-13.
20. Alford DP, LaBelle CT, Kretsch N, et al. Collaborative care of opioid-addicted patients in primary care using buprenorphine: five-year experience. *Archives of internal medicine.* 2011;171(5):425-431.
21. Pettinati H, Weiss R, Miller W, Donovan D, Rounsaville B. Medical Management Treatment Manual Unpublished manual. COMBINE. National Institute on Alcohol Abuse and Alcoholism, Bethesda, MD.; 2000.
22. Fiellin DA, Pantalon MV, Chawarski MC, et al. Counseling plus buprenorphine-naloxone maintenance therapy for opioid dependence. *The New England journal of medicine.* 2006;355(4):365-374.
23. Springer SA, Altice FL, Herme M, Di Paola A. Design and methods of a double blind randomized placebo-controlled trial of extended-release naltrexone for alcohol dependent and hazardous drinking prisoners with HIV who are transitioning to the community. *Contemp Clin Trials.* 2014;37(2):209-218.
24. Di Paola A, Lincoln T, Skiest DJ, Desabrais M, Altice FL, Springer SA. Design and methods of a double blind randomized placebo-controlled trial of extended-release naltrexone for HIV-infected, opioid dependent prisoners and jail detainees who are transitioning to the community. *Contemp Clin Trials.* 2014;39(2):256-268.
25. (http://www.orasure.com/products-infectious/products-infectious.asp).
26. Branson BM, Handsfield HH, Lampe MA, et al. Revised recommendations for HIV testing of adults, adolescents, and pregnant women in health-care settings. *MMWR. Recommendations and reports : Morbidity and mortality weekly report. Recommendations and reports / Centers for Disease Control.* 2006;55(RR-14):1-17; quiz CE11-14.
27. Sobell L, Sobell M. Timeline followback: a technique for assessing self-reported ethanol consumption. In: J A, RZ L, eds. *Measuring Alcohol Consumption: Psychosocial and Biological Methods*. Totowa, NJ: Humana Press; 1992:41-72.
28. Sobell MB, Sobell LC. An excellent springboard. *Addiction.* 2000;95(11):1712-1715.
29. Sheehan DV, Lecrubier Y, Sheehan KH, et al. The Mini-International Neuropsychiatric Interview (M.I.N.I.): the development and validation of a structured diagnostic psychiatric interview for DSM-IV and ICD-10. *The Journal of clinical psychiatry.* 1998;59 Suppl 20:22-33;quiz 34-57.
30. Saunders JB, Aasland OG, Babor TF, de la Fuente JR, Grant M. Development of the Alcohol Use Disorders Identification Test (AUDIT): WHO Collaborative Project on Early Detection of Persons with Harmful Alcohol Consumption--II. *Addiction (Abingdon, England).* 1993;88(6):791-804.
31. Kroenke K, Spitzer RL, Williams JB. The PHQ-9: validity of a brief depression severity measure. *J Gen Intern Med.* 2001;16(9):606-613.
32. Spitzer RL, Kroenke K, Williams JB. Validation and utility of a self-report version of PRIME-MD: the PHQ primary care study. Primary Care Evaluation of Mental Disorders. Patient Health Questionnaire. *JAMA.* 1999;282(18):1737-1744.
33. Blevins CA, Weathers FW, Davis MT, Witte TK, Domino JL. The Posttraumatic Stress Disorder Checklist for DSM-5 (PCL-5): Development and Initial Psychometric Evaluation. *Journal of traumatic stress.* 2015;28(6):489-498.
34. Kessler RC, Adler L, Ames M, et al. The World Health Organization Adult ADHD Self-Report Scale (ASRS): a short screening scale for use in the general population. *Psychological medicine.* 2005;35(2):245-256.
35. World Health Organization Quality of Life Group. Development of the World Health Organization WHOQOL-BREF quality of life assessment. *Psychology and Medicine.* 1998;28:351-558.

36. van Walraven C, Austin PC, Jennings A, Quan H, Forster AJ. A modification of the Elixhauser comorbidity measures into a point system for hospital death using administrative data. *Medical care.* 2009;47(6):626-633.
37. Krebs EE, Lorenz KA, Bair MJ, et al. Development and initial validation of the PEG, a three-item scale assessing pain intensity and interference. *J Gen Intern Med.* 2009;24(6):733-738.
38. Feldhaus KM, Koziol-McLain J, Amsbury HL, Norton IM, Lowenstein SR, Abbott JT. Accuracy of 3 brief screening questions for detecting partner violence in the emergency department. *Jama.* 1997;277(17):1357-1361.
39. Ramsay MA, Savege TM, Simpson BR, Goodwin R. Controlled sedation with alphaxalone-alphadolone. *British medical journal.* 1974;2(5920):656-659.
40. Levine J, Schooler NR. SAFTEE: a technique for the systematic assessment of side effects in clinical trials. *Psychopharmacol Bull.* 1986;22(2):343-381.
41. Wickersham JA, Azar MM, Cannon CM, Altice FL, Springer SA. Validation of a Brief Measure of Opioid Dependence: The Rapid Opioid Dependence Screen (RODS). *J Correct Health Care.* 2015;21(1):12-26.
42. D'Onofrio G, O'Connor PG, Pantalon MV, et al. Emergency department-initiated buprenorphine/naloxone treatment for opioid dependence: a randomized clinical trial. *Jama.* 2015;313(16):1636-1644.
43. Liebschutz JM, Crooks D, Herman D, et al. Buprenorphine treatment for hospitalized, opioid-dependent patients: a randomized clinical trial. *JAMA Intern Med.* 2014;174(8):1369-1376.
44. Hser YI, Huang D, Saxon AJ, et al. Distinctive Trajectories of Opioid Use Over an Extended Follow-up of Patients in a Multisite Trial on Buprenorphine + Naloxone and Methadone. *J Addict Med.* 2017;11(1):63-69.
45. Weiss RD, Potter JS, Fiellin DA, et al. Adjunctive counseling during brief and extended buprenorphine-naloxone treatment for prescription opioid dependence: a 2-phase randomized controlled trial. *Arch Gen Psychiatry.* 2011;68(12):1238-1246.
46. Weiss RD, Potter JS, Griffin ML, et al. Long-term outcomes from the National Drug Abuse Treatment Clinical Trials Network Prescription Opioid Addiction Treatment Study. *Drug Alcohol Depend.* 2015;150:112-119.
47. Little RJA, Rubin DB. *Statistical Analysis with Missing Data, 2nd Edition.* 2002.
48. Taylor AB, MacKinnon DP, Tein Y. Tests of the Three-Path Mediated Effect. *Organizational Research Methods.* 2008;11(2):241-269.

19.0 Appendix A

COMMIT Covid-19 Appendix

The following procedures will be employed if there are research restrictions in place regarding the covid-19 pandemic. Given the unpredictable nature of the pandemic going forward, these procedures may be employed at different times and/or at different sites, all in accordance with both MUSC IRB and local institutional guidelines. All research procedures such as consent, baseline assessments and follow-up assessments will be guided by the principle of minimizing non-clinical research staff exposure time to participants with known, suspected or possible covid-19 with the goal of assuring safety for both participants and research staff.

Remote Consent

The following remote consent methods will be utilized as recommended per MUSC IRB and FDA guidance on consent processes during the covid-19 public health emergency (https://research.musc.edu/resources/sctr/research-resources/remote-and-virtual-trials, Study Team Guidance; https://www.fda.gov/regulatory-information/search-fda-guidance-documents/fda-guidance-conduct-clinical-trials-medical-products-during-covid-19-public-health-emergency). Doxy.me is an e-consent platform acceptable to guidelines outlined by MUSC IRB. Doxy.me will be only be used at local sites in which their institutions also approve of its use. It will be used when in-person consent not possible (e.g. for an inpatient with covid-19) altering the risk benefit of entering the patient

room. The previously existing Informed Consent Form will be utilized. For this platform, participants will need a smartphone. If they do not have one an alternate remote consent process will be used. The participant will be directed through the consent process over the telephone and doxy.me will be utilized to obtain the final signature. Alternatively, the participant may follow along on the doxy.me platform and review the ICF while it is being explained over the telephone and then sign the ICF via the electronic platform. A copy of the signed consent form will be provided to the participant via e-mail.
If e-consent is not possible in a situation where the direct consent process is not feasible, alternate pathways have been identified, as excerpted below from the FDA guidance referenced above:

> "If direct communication with the patient in isolation is not feasible or safe, the investigator (or their designee) obtains the patient's phone number and arranges a threeway call or video conference with the patient, an impartial witness and, if desired and feasible, additional participants requested by the patient (e.g., next of kin) 3. To ensure that patients are approached in a consistent fashion, a standard process should be used that will accomplish the following:
> O Identification of who is on the call
> o Review of the informed consent with the patient by the investigator (or their designee) and response to any questions the patient may have
> o Confirmation by the witness that the patient's questions have been answered
> o Confirmation by the investigator that the patient is willing to participate in the trial and sign the informed consent document while the witness is listening on the phone
> o Verbal confirmation by the patient that they would like to participate in the trial and that they have signed and dated the informed consent document that is in their possession If the signed informed consent document cannot be collected from the patient's location and included in the study records, FDA considers the following two options acceptable to provide documentation that the patient signed the informed consent document:
> • A dated attestation by the witness who participated in the call and by the investigator that the patient confirmed that they agreed to participate in the study and signed the informed consent.
> OR
> • A photograph of the informed consent document with attestation by the person entering the photograph into the study record that states how that photograph was obtained and that it is a photograph of the informed consent signed by the patient. A copy of the informed consent document signed by the investigator and witness should be placed in the patient's trial source documents, with a notation by the investigator of how the consent was obtained (e.g., telephone call). The trial record at the investigational site should document how it was confirmed that the patient signed the consent form (i.e., either using attestation by the witness and investigator or the photograph of the signed consent). The note should include a statement of why the informed consent document signed by the patient was not retained (e.g., due to potential contamination of the document by infectious material)."

The potential participant will be given an enrollment packet at the beginning of the consent process that will include a copy of the informed consent along with an emergency contact card and a calendar which will be used for the baseline study visit if the patient consents to participate in the study. The patient will retain the copy of the informed consent that they reviewed with the study team.

Remote Study Assessments
All research activities will be conducted in a manner that is line with institutional guidelines regarding Infection Prevention and covid-19 mitigation. This includes following guidance regarding use of personal protective equipment in conducting inpatient study assessments in participants with covid-19. In-person assessments will take place based on the regulations and restrictions of each individual institution based on the nature of the SARS-CoV2 pandemic. If an in-person study assessment is restricted or felt to have an unfavorable risk/benefit profile, the following remote study assessment methods will be applied.
<u>Inpatient</u>

Inpatient baseline and follow-up study assessments can be performed remotely via two processes. All non-clinical assessments in the current study visit schedule can be conducted over the telephone. If a participant does not have a personal telephone, the hospital room phone may be used. Clinical

assessments (COWS, Ramsay) can be performed in collaboration with inpatient primary healthcare team (physician, nurse). Both the COWS and Ramsay are common clinical assessments that can be written as nursing order as part of standard of care for management of opioid use disorder. The other clinical assessments (medication reconciliation, Substance Use Treatment Form, Elixhauser Comorbidity Index and ID Questionnaire) can be performed over the phone and through chart review. The method of assessment will be documented in the research database.

Ambulatory

If a participant is placed in isolation due to diagnosis of covid-19 or placed in quarantine due to a contact with a known positive, we will employ remote study assessment methods such that study activities may continue. All non-clinical assessments in the study visit schedule can be performed over the telephone. For participants who do not have a telephone, we will assist them in enrolling in programs such as Safelink Wireless and Assurance Wireless which are federal/state programs that can facilitate phone service for those who qualify. If remote visits are not possible, participants will be seen either in research clinical space or ambulatory direct clinical spaces with appropriate PPE and physical distancing. Clinical assessments and study drug administration will take place in these settings as well, as per current visit schedule.

Laboratory Assessments

Baseline point of care assessments (HCV, HIV, urine drug screen, and urine pregnancy tests) have been broadened to allow for hospital based assays to be used if needed which removes the need for research personnel to enter the room and collect baseline samples.

COMMIT Telephone Remote Consent Process

The following procedures will be employed if conducting the informed consent process in person is not feasible. These procedures may be employed at different times and/or at different sites, all in accordance with both MUSC IRB and local institutional guidelines. This alternative consenting process may be used to enroll patients at sites which require a study clinician to conduct the consenting process. To conduct telephone consents, three study team members will need to be involved—one staff member to be present with the patient and two staff who will be off-site:

- The patient will be given a physical copy of the Informed Consent Form (ICF) ahead of the planned telephone consent process to ensure the patient has ample time to review the document and to prepare any questions
- On the day of planned consent, a local site staff research team member will meet with the patient and serve as a witness to the consenting process.
- The study staff member who is physically present with the patient will conference call the study clinician, who will not be physically present, and the study clinician will conduct the informed consent process over the phone using their own copy of the ICF, while the patient follows along with their own copy of the ICF.
- An additional study team member will be present with the study clinician, to serve as an additional witness to the consenting process.
- If the patient wishes to enroll in the trial, the study clinician will remotely administer the Informed Consent Quiz to ensure patient understanding of the trial.
- The participant will then sign their copy ICF where applicable, with the assistance of the local study team member.
- The local study staff member present with the patient will also sign this ICF as a witness.
    - The study staff member will then securely send the signed ICF to the study clinician, via secure file transfer, who will also sign the ICF, and send back to the study staff member
    - The study participant will receive a copy of the ICF containing their signature and the clinician signature
    - All signed copies of the ICF will be retained in the patient's study chart

- The study clinician and additional study staff member will both sign the Alternative Consenting Documentation Form indicating that consent was conducted remotely, which will also be filed in the patient’s chart.
