# Supplementary material for: Initiating Injectable Buprenorphine in People Hospitalized With Infections: A Randomized Clinical Trial
Source: JAMA Netw Open. 2025 May 30;8(5):e2513000. doi: 10.1001/jamanetworkopen.2025.13000 (PMC12125644; doi:10.1001/jamanetworkopen.2025.13000)
Supplement: Supplement 2. — eTable 1. Week 12 Outcomes eTable 2. Week 24 Outcomes eTable 3. Interview Retention [file jamanetwopen-e2513000-s002.pdf]

Supplementary Online Content

Seval N, Roth P, Frank CA, et al. Initiating injectable buprenorphine in people hospitalized with infections: a randomized clinical trial. *JAMA Netw Open*. 2025;8(5):e2513000. doi:10.1001/jamanetworkopen.2025.13000

eTable 1. Week 12 Outcomes

eTable 2. Week 24 Outcomes

This supplementary material has been provided by the authors to give readers additional information about their work.

© 2025 Seval N et al. *JAMA Network Open*.

eTable 1. Week 12 Outcomes

**Week 12 Outcomes**	**Parameter**	**Level**	**Estimate**	**StdErr**	**ProbChiSq**
Primary Enrollment in MOUD	Intercept		-0.20	0.15	0.173
	SiteID	Penn	0.10	0.32	0.746
	SiteID	Prisma	0.00	0.13	0.979
	**Arm**	**ID**	**0.01**	**0.13**	**0.940**
	pre_moud_prescrip_ba	No	-0.50	0.12	0.000
Index Infection Treatment Completed	Intercept		-0.10	0.08	0.192
	SiteID	Penn	-0.28	0.24	0.249
	SiteID	Prisma	-0.14	0.09	0.110
	**Arm**	**ID**	**-0.04**	**0.07**	**0.624**
	pre_moud_prescrip_ba	No	0.00	0.09	0.983
Index Infection Cured	Intercept		-2.24	0.53	0.000
	SiteID	Penn	0.03	0.68	0.968
	SiteID	Prisma	-0.37	0.38	0.330
	**Arm**	**ID**	**0.42**	**0.36**	**0.233**
	pre_moud_prescrip_ba	No	0.57	0.52	0.266
HCV Detectable Viral Load	Intercept		-1.69	0.33	0.000
	SiteID	Penn	-2.28	3.22	0.479
	SiteID	Prisma	0.61	0.25	0.017
	**Arm**	**ID**	**-0.17**	**0.19**	**0.380**
	pre_moud_prescrip_ba	No	-0.12	0.21	0.560
	HCVVL_cat_base	No	1.08	0.22	0.000
HIV Risk - Condomless Sex	Intercept		-1.02	0.23	0.000
	SiteID	Penn	0.28	0.58	0.629
	SiteID	Prisma	0.11	0.17	0.511
	**Arm**	**ID**	**0.08**	**0.13**	**0.528**
	pre_moud_prescrip_ba	No	0.13	0.20	0.523
	VagSex_base	No	0.53	0.17	0.002
HIV Risk – Shared works*	Intercept		8.06	98.06	0.934
	**Arm**	**ID**	**0.15**	**0.53**	**0.773**
	pre_moud_prescrip_ba	No	-5.40	98.06	0.956
	ShareWorks_base	No	0.89	0.60	0.137
WHO QoL	Intercept		64.99	7.03	0.000
	SiteID	Penn	5.58	6.81	0.413
	SiteID	Prisma	-1.03	3.04	0.735
	**Arm**	**ID**	**3.16**	**2.64**	**0.231**
	pre_moud_prescrip_ba	No	-1.25	3.24	0.699
	who_score_base		0.26	0.09	0.003
**Week 12 Outcomes continued**	**Parameter**	**Level**	**Estimate**	**StdErr**	**ProbChiSq**

© 2025 Seval N et al. *JAMA Network Open*.

Pain (PEG)	Intercept		2.97	0.80	0.000
	SiteID	Penn	-1.11	1.32	0.399
	SiteID	Prisma	-0.87	0.59	0.145
	**Arm**	**ID**	**-0.84**	**0.51**	**0.098**
	pre_moud_prescrip_ba	No	0.69	0.63	0.271
	Pain_base		0.14	0.09	0.120
PHQ9	Intercept		1.70	1.86	0.359
	SiteID	Penn	-1.39	2.79	0.618
	SiteID	Prisma	1.48	1.24	0.234
	**Arm**	**ID**	**-0.77**	**1.07**	**0.471**
	pre_moud_prescrip_ba	No	0.94	1.32	0.477
	depression_score_bas		0.40	0.08	0.000
TLFB days of reported use	Intercept		3.67	2.99	0.220
	SiteID	Penn	1.30	4.77	0.786
	SiteID	Prisma	-1.38	2.34	0.556
	**Arm**	**ID**	**-1.71**	**2.04**	**0.402**
	pre_moud_prescrip_ba	No	8.01	2.74	0.004
	tlfbdays_ap_op_base		0.21	0.10	0.041
Negative Urine Tox for Opioids	Intercept		-1.09	0.24	0.000
	SiteID	Penn	-0.39	0.36	0.272
	SiteID	Prisma	-0.11	0.12	0.371
	**Arm**	**ID**	**0.16**	**0.12**	**0.188**
	pre_moud_prescrip_ba	No	0.26	0.17	0.135
	urineTox_opi_base_p	Neg	0.51	0.17	0.002

© 2025 Seval N et al. *JAMA Network Open*.

eTable 2. Week 24 Outcomes

**Week 24 Outcomes**	**Parameter**	**Level1**	**Estimate**	**StdErr**	**ProbChiSq**
Primary Enrollment in MOUD	Intercept		-0.28	0.13	0.038
	SiteID	Penn	0.28	0.36	0.437
	SiteID	Prisma	-0.11	0.17	0.500
	**Arm**	**ID**	**-0.07**	**0.16**	**0.668**
	pre_moud_prescrip_ba	No	-0.57	0.17	0.001
Index Infection Treatment Completed	Intercept		-0.10	0.08	0.192
	SiteID	Penn	-0.28	0.24	0.249
	SiteID	Prisma	-0.14	0.09	0.110
	**Arm**	**ID**	**-0.04**	**0.07**	**0.624**
	pre_moud_prescrip_ba	No	0.00	0.09	0.983
Index Infection Cured	Intercept		-2.24	0.53	0.000
	SiteID	Penn	0.03	0.68	0.968
	SiteID	Prisma	-0.37	0.38	0.330
	**Arm**	**ID**	**0.42**	**0.36**	**0.233**
	pre_moud_prescrip_ba	No	0.57	0.52	0.266
HIV Risk - Condomless Sex	Intercept		-0.75	0.22	0.001
	SiteID	Penn	0.18	0.28	0.525
	SiteID	Prisma	0.14	0.16	0.382
	Arm	ID	-0.01	0.14	0.967
	pre_moud_prescrip_ba	No	-0.08	0.16	0.626
	VagSex_base	No	0.43	0.17	0.012
HIV Risk – Shared works	Intercept		8.35	120.43	0.945
	**Arm**	**ID**	**0.57**	**0.63**	**0.371**
	pre_moud_prescrip_ba	No	-5.53	120.43	0.963
	ShareWorks_base	No	0.52	0.64	0.412
WHO QoL	Intercept		61.91	7.59	0.000
	SiteID	Penn	-0.05	6.78	0.994
	SiteID	Prisma	1.36	3.10	0.661
	**Arm**	**ID**	**3.59**	**2.75**	**0.191**
	pre_moud_prescrip_ba	No	-5.48	3.42	0.109
	who_score_base		0.33	0.09	0.000
Pain (PEG)	Intercept		1.63	0.85	0.056
	SiteID	Penn	0.04	1.36	0.976
	SiteID	Prisma	-0.49	0.63	0.431
	**Arm**	**ID**	**-0.49**	**0.55**	**0.370**
	pre_moud_prescrip_ba	No	0.90	0.69	0.190
	Pain_base		0.25	0.10	0.009
ADHD	Intercept		-0.49	0.19	0.008
	SiteID	Penn	-0.15	0.40	0.699

© 2025 Seval N et al. *JAMA Network Open*.

**Week 24 Outcomes continued**	**Parameter**	**Level1**	**Estimate**	**StdErr**	**ProbChiSq**
	SiteID	Prisma	0.01	0.22	0.970
	**Arm**	**ID**	**0.04**	**0.15**	**0.790**
	pre_moud_prescrip_ba	No	-0.26	0.26	0.321
	ProvisionalDx_base	0	0.44	0.21	0.034
PHQ9	Intercept		-0.57	1.84	0.759
	SiteID	Penn	2.23	2.58	0.386
	SiteID	Prisma	0.62	1.16	0.590
	**Arm**	**ID**	**-0.86**	**1.03**	**0.402**
	pre_moud_prescrip_ba	No	2.67	1.29	0.038
	depression_score_bas		0.50	0.08	0.000
TLFB days of reported use	Intercept		7.38	3.15	0.019
	SiteID	Penn	2.65	5.02	0.597
	SiteID	Prisma	0.44	2.47	0.857
	**Arm**	**ID**	**-1.07**	**2.15**	**0.619**
	pre_moud_prescrip_ba	No	3.94	2.89	0.173
	tlfbdays_ap_op_base		0.28	0.11	0.009
Negative Urine Tox for Opioids	Intercept		-1.25	0.25	0.000
	SiteID	Penn	-0.27	0.36	0.449
	SiteID	Prisma	-0.01	0.13	0.930
	**Arm**	**ID**	**0.15**	**0.11**	**0.174**
	pre_moud_prescrip_ba	No	0.47	0.22	0.030
	urineTox_opi_base_p	0	0.41	0.15	0.006

© 2025 Seval N et al. *JAMA Network Open*.
