# Supplementary material for: Initiating Injectable Buprenorphine in People Hospitalized With Infections: A Randomized Clinical Trial
Source: JAMA Netw Open. 2025 May 30;8(5):e2513000. doi: 10.1001/jamanetworkopen.2025.13000 (PMC12125644; doi:10.1001/jamanetworkopen.2025.13000)
Supplement: Supplement 3. — Data Sharing Statement [file jamanetwopen-e2513000-s003.pdf]

Data Sharing Statement

Seval. Initiating Injectable Buprenorphine in People Hospitalized With Infections. *JAMA Netw Open*. Published May 30, 2025. doi:10.1001/jamanetworkopen.2025.13000

Data
Additional Information: ClinicalTrials.gov identifier: NCT04180020
Data available: Yes
Data types: Deidentified participant data, Data dictionary
How to access data: sandra.springer@yale.edu
When available: With publication

Supporting Documents
Document types: Statistical/analytic code, Informed consent form
How to access documents: sandra.springer@yale.edu
When available: With publication

Additional Information
Who can access the data: researchers whose proposed use of the data has been approved
Types of analyses: related to the research
Mechanisms of data availability: after approval of the proposal and signed data access agreement with investigator support
